# Associations of Social and Demographic Factors on the Outcomes of Ocular Melanoma and Other Adult Ocular Neoplasms in the United States: A Systematic Review

**DOI:** 10.1002/cesm.70075

**Published:** 2026-03-10

**Authors:** Daniel Shaughnessy, Vijay Joshi, Natalia Dellavalle, Louis Leslie, Michael Edwards, Timothy Waxweiler, Tianjing Li, Riaz Qureshi

**Affiliations:** ^1^ University of Colorado Anschutz Medical Campus Aurora Colorado USA

**Keywords:** cancer, ocular melanoma, ocular neoplasms, social determinants of health

## Abstract

**Introduction:**

Social determinants of health (SDOH), including economic stability, education access and quality, healthcare access and quality, neighborhood and built environment, and social and community context, shape gaps in health outcomes across many conditions. Ocular neoplasms are no exception. Cancers such as uveal melanoma, conjunctival squamous cell carcinoma, ocular lymphoma, and ocular Kaposi sarcoma may be especially vulnerable to social and demographic influences. We systematically reviewed documented associations between SDOH and these ocular cancers in the United States.

**Methods:**

Following a pre‐registered protocol, we searched MEDLINE, Embase, and Web of Science (from January 2000 to November 2023) for primary studies of any design that evaluated one or more relationships between SDOH and outcomes related to the ocular cancers listed above. Outcomes included cancer incidence, stage at diagnosis, treatment patterns, survival, and mortality. We extracted study design, population, exposure, and outcome characteristics, classified each exposure‐outcome association by its direction (e.g., favorable, unfavorable, or null), and assessed the risk of bias using a modified Newcastle‐Ottawa Scale. Due to heterogeneity in exposure and outcome definitions, we narratively synthesized findings by SDOH domain.

**Results:**

We included 21 studies examining 167 unique associations. Social and community context, typically represented as race and ethnicity, was the most frequently studied domain, followed by economic stability (e.g., income) and healthcare access and quality (e.g., insurance type or travel distance). Across domains, lower socioeconomic status, public or no insurance, minority racial and ethnic identity, and care at academic centers generally are associated with later stage at diagnosis, higher odds of enucleation, or worse survival. Higher income, private insurance, and treatment at experienced facilities were often associated to earlier presentation and better outcomes.

**Conclusion:**

SDOH have a measurable and often unfavorable relationship with the diagnosis, management, and prognosis of rare adult ocular cancers in the United States. Standardized SDOH exposures and measurements, prospective data collection, and adjustment for confounding are necessary to strengthen the evidence and guide multi‐domain interventions (e.g., expanded insurance, travel assistance to high‐volume centers, and community eye‐health initiatives) aimed at narrowing these gaps.

## Summary

Not everyone with eye cancer in the United States receives the same quality of care. This review examined how social and economic factors affect survival and treatment outcomes for people with rare eye cancers (including uveal melanoma, ocular lymphoma, conjunctival squamous cell carcinoma, and ocular Kaposi sarcoma) by bringing together findings from 21 studies.

We found that patients with lower incomes, less education, or public or no health insurance were more likely to be diagnosed at a later stage, more likely to have their eye surgically removed, and less likely to survive. People from racial or ethnic minority groups faced similar disadvantages. In contrast, patients with higher incomes and private insurance tended to be diagnosed earlier and have better chances of recovery.

These findings make clear that where you start in life can shape where you end up in your cancer journey and that this is not inevitable. Improving access to health insurance, helping patients navigate their way to specialist care centers, and investing in community health education could all help close these gaps. People with eye cancer deserve the same chance of early diagnosis and effective treatment, regardless of their income, insurance status, or background.

## 1 Introduction

Understanding how societal, economic, and demographic factors interact and are associated with health outcomes is essential for effective prevention and public health strategies [1, 2]. Moreover, examining how these factors contribute to unequal outcomes is key to enhancing the efficiency and effectiveness of clinical care [1, 2]. These factors, collectively known as social determinants of health (SDOH), are widely recognized in the United States and globally as critical influences on individual and population health. SDOH encompass five primary domains according to the Healthy People 2030 framework [1]: economic stability, education access and quality, healthcare access and quality, neighborhood and built environment, and social and community context. Each domain includes numerous exposures and attributes that directly affect the care individuals receive and their health outcomes across a wide range of conditions [1].

In this systematic review, our objective was to assess the relationship between SDOH and a variety of ocular cancers, including uveal melanoma, conjunctival squamous cell carcinoma, ocular lymphoma, and ocular Kaposi sarcoma in the US. We cover the relationships between SDOH and retinoblastoma, the most common ocular cancer in children, in a separate review submitted simultaneously. Uveal melanoma is the most frequently studied ocular cancer in this review, and is the most common primary intraocular malignancy in adults [3, 4]. Uveal melanoma arises from melanocytes in the uveal tract (most often the choroid) [5, 6]. Modern treatment can include radiotherapy, phototherapy, and surgical interventions, with enucleation reserved for larger tumors [7]. About 50% of patients develop metastatic disease, often to the liver, which is associated with high mortality [4, 5, 7]. The other three cancers, ocular lymphoma, ocular Kaposi sarcoma, and squamous cell carcinoma of the conjunctiva, are often associated with HIV/AIDS. We included the studies that looked at these relationships.

## 2 Methods

We performed this review within a set of systematic reviews done with assistance from Cochrane Eyes and Vision US Project (CEV@US) to examine various aspects of eye health and their relation to the SDOH [8]. In this review, we focused on all ocular cancers, excluding retinoblastoma, and followed a protocol published on Open Science Framework [9]. Appendix A contains a detailed description of our methods, summarized below, and Appendix B presents the data extraction form.

We identified eligible studies from a master database on social determinants of ocular health in the US. The master database was developed and maintained by CEV@US, and it was constructed by searching MEDLINE, Embase, and Web of Science Core Collection from year 2000 to present. This date range was chosen due to the substantial shifts of US demographics and social constructs related to SDOH that have occurred over the past two decades. We screened full texts of the records tagged for ocular cancers. We excluded studies on retinoblastoma from this review as we explored them in a separate paper. Given the relatively large number of studies related to SDOH and retinoblastoma specifically, it would have been impractical to additionally discuss the findings related to retinoblastoma in this review. We also searched the reference list of included studies for relevant manuscripts. We included primary studies that examined the relationship between any domain of SDOH and any ocular cancers. We did not restrict studies based on their design.

We extracted characteristics of the study, population, exposures, outcomes, and estimates of the associations using Systematic Review Data Repository (SRDR + ) and Qualtrics [8, 10]. We assessed the risk of bias using a modified Newcastle‐Ottawa Scale. We employed a single assessor plus verification approach for both data extraction and risk of bias, resolving discrepancies through discussion or adjudication with a third reviewer.

Due to the heterogeneity of the studies and analyses, including variations in definitions of exposures, outcomes, and measures of association, we were unable to conduct any meta‐analyses (i.e., quantitative syntheses). We provided a narrative synthesis of the associations organized by SDOH domain. We created data displays to portray the breadth and types of associations.

We classified all association estimates according to their directionality following the guidance in Chapter 13 of the Cochrane Handbook on “synthesis using other methods” [11]. Association directionality was classified as follows: favorable if a “worse” exposure compared with “better” exposure (e.g., low income vs. high income), was associated with improved outcomes (e.g., reduced incidence); unfavorable if a “worse” exposure was associated with worse outcomes (e.g., increased mortality); and null if no clear relationship was observed. To standardize the classification of directionality, it was sometimes necessary to invert the association from its original assessment. For example, if a study found that higher income levels were associated with decreased mortality when compared with lower income levels, we classified this association as “unfavorable” because a positive association for the better exposure represents a negative association for the worse exposure. In some cases, the directionality of the exposure‐outcome relationship could not be determined, such as when there was no clearly defined reference group; we labeled these associations “NA.”

Given the large number of associations across studies, we were unable to describe all estimates in the text when synthesizing the evidence. Instead, we highlighted those with a substantial effect size and focused on summarizing patterns, as well as any findings that contradicted expected trends, regardless of statistical significance.

## 3 Results

**FIGURE 1:**
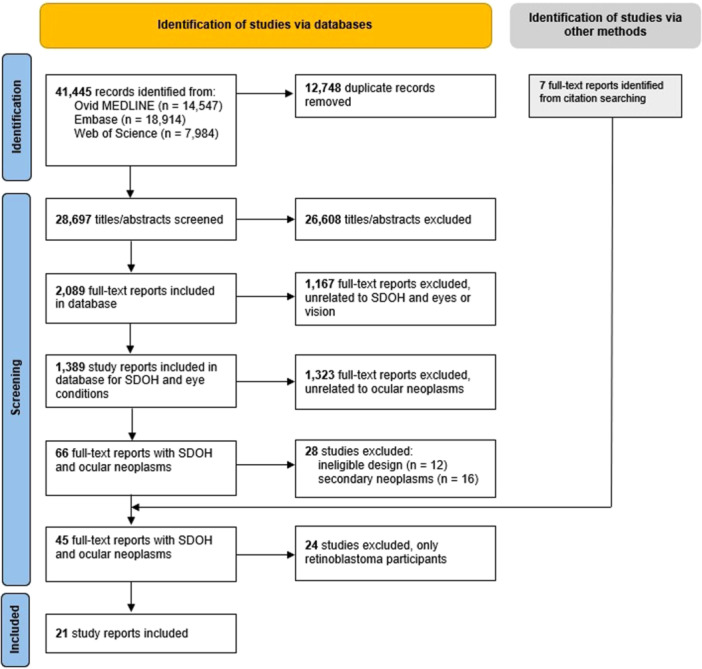
PRISMA flowchart for systematic review.

As of November 30, 2023, the master database contained 1389 reports related to SDOH and eye health, 66 of which were categorized under ocular neoplasms and were further assessed for this review. We included 38 records from the database and identified seven additional reports by searching the reference list of included studies. Of the 45 eligible studies related to ocular cancers, 21 described associations with uveal melanoma, squamous cell carcinoma of the conjunctiva, ocular lymphoma, and ocular Kaposi sarcoma, which are included in this review (Figure 1). The other 24 papers were specific to retinoblastoma, which are included in a separate review.

### 3.1 Characteristics of Included Studies

Sixteen cohort studies, three retrospective chart reviews, and two reports from randomized controlled trials published after the year 2000 were included; data for the studies were collected from 1969 to 2021 (Table 1). Many studies were based on secondary data analyses of previously established cancer registries and databases, including the Surveillance, Epidemiology, and End Results Program (SEER) (*n* = 7), the National Cancer Database (*n* = 3), and two reports utilized a dataset from the Collaborative Ocular Melanoma Study (*n* = 2). Four studies examined institution‐specific patient populations (*n* = 4), while four others utilized other statewide or national datasets, including the Ohio Cancer Incidence Surveillance System, the North American Association of Central Cancer Registries, the US HIV/AIDS Cancer Match study, and the NIH‐AARP Diet and Health Study.

**TABLE 1:** Summary of selected characteristics of included studies.

Study	Study years	Study design	Study population (representativeness)	Sample size	Participants' characteristics	SDOH domain studied (role)	Ocular neoplasm measure (role)	Study findings	Funding source
Alfaar 2022	1973 to 2017	Cohort study	Individuals diagnosed as having uveal melanoma between 1973 and 2017 and identified in the SEER 18 database (national)	10,557	Age group 0–39: 8% 40–59: 35% 60–80+: 57% Female: 48% White: 98% Hispanic: NR	Healthcare access and quality; Social and community context (exposure)	Uveal melanoma ‐ survival (outcome)	The study found better 10‐year survival in uveal melanoma patients who maintained their marriages compared to unmarried, divorced, and widowed patients.	None
Chevli 2022	2004 to 2015	Cohort study	Patients found from the National Cancer Database that were 18 years or older with a histologically confirmed diagnosis of uveal melanoma between 2004 and 2015 without nodal disease or distant metastasis at diagnosis (national)	15,662	Age ≤ 60: 42% > 60: 58% Female: 48% White: 95% Hispanic: (NR)	Economic stability; Education access and quality; Healthcare access and quality; Neighborhood and built environment (exposure)	Small/medium uveal melanoma ‐ Enucleation (outcome)	The study found significant associations with exposures across all SDOH domains with the likelihood of receiving enucleation treatment for uveal melanoma compared to brachytherapy.	NR
Choudhry 2023	2006 to 2017	Cohort study	Patient diagnosed as having primary uveal melanoma, conjunctival melanoma, or retinoblastoma between January, 2006 and December, 2017, treated with curative intent, and identified in the National Cancer Database (national)	4800	Median age at diagnosis: 64 Female: 47% White: 92% Hispanic: NR	Social and community context, Healthcare access and quality, Economic stability (exposure)	Uveal melanoma, conjunctival melanoma, retinoblastoma ‐ advanced clinical tumor presentation (outcome) and 5‐year survival (outcome)	The study did a combined analysis but found having insurance and lower income were both significantly associated with worse ocular cancer staging on presentation.	Research to Prevent Blindness Inc. and the Wilmer Eye Institute
Clevenger 2023	2000 to 2019	Cohort study	Patients diagnosed with uveal melanoma between 2000 and 2019 and identified in the Ohio Cancer Incidence Surveillance System (state)	1617	Average age (SD): NR (NR) Female: NR White: NR Hispanic: NR	Economic stability; Education access and quality; Healthcare access and quality; Social and community context (exposure)	Uveal melanoma ‐ incidence (outcome)	The study found higher incidence of uveal melanoma in rural counties, low‐education counties, counties with higher average income, and in counties with lower proportion of minority populations.	The National Institute of Health, Research to Prevent Blindness Inc., and Cleveland Eye Bank Foundation
COMS 2001	1986 to 1998	RCT	Patients diagnosed as having ocular melanoma between 1986 and 1998 in both the US and Canada (national)	6906	Medium tumor trial Age < 60: 46% ≥ 60: 54% Female: 49% White: NR Hispanic: NR Large tumor trial Age < 60: 44% ≥ 60: 56% Female: 43% White: NR Hispanic: NR	Healthcare access and quality (exposure)	Enrollment in tumor trial (outcome)	The study found evidence that patients who resided in the same state as a clinical center running a research trial increased the likelihood that the patient would enroll in a research trial at that facility.	The National Eye Institute and the National Cancer Institute
COMS 2004	1986 to 2000	RCT	Patients with ocular melanoma that were free of metastasis or other cancers from the US and Canada recruited between 1986 and 1994 (national)	1003	Large tumor trial Age ≤ 60: 46% > 60: 54% Female: 42% White: NR Hispanic: NR	Education access and quality (exposure)	Metastatic choroidal melanoma ‐ 5 and 10 year mortality (outcomes)	The study found a non‐significant decrease in the risk of 5‐year mortality for patients with metastatic ocular melanoma among patients with more than a high school education when compared to patients with less than a high school education.	The National Eye Institute, National Cancer Institute, National Institutes of Health, and U. S. Department of Health and Human Services
Davanzo 2019	2014 to 2015	Cohort study	Patients diagnosed as having uveal melanoma and treated at the Cleveland Clinic Institute from January 2014 to June 2015 (institutional).	107	Average age (SD): 61.4 (13.8) Female: NR White: NR Hispanic: NR	Social and community context, Economic stability, Healthcare access and quality (exposure)	Uveal melanoma ‐ risk of metastasis (outcome)	The study found no difference in the distribution of insurance types or distance to clinic between patients with a high risk of metastasis and those with low risk of metastasis of ocular melanoma.	None
Emmanuel 2012	1995 to 2006	Cohort study	Patients diagnosed as having ocular cancer from 1995 to 2006 from six US states and two metropolitan areas (national)	178	Age ≤ 60: 36% Age > 60: 64% Female: 40% White: 91% Hispanic: 2%	Social and community context (exposure)	All eye neoplasms ‐ incidence (outcome)	The study found no differences between incidence rates of eye cancers among different races.	Intramural Research Program of the National Institutes of Health and the National Cancer Institute
Guech‐Ongey 2008	1996 to 2004	Cohort study	Adults with eye cancers (aged > 15 or older) in 9 US states and 5 metropolitan areas diagnosed with AIDS from 1980 to 2004 (national)	67	Age at AIDS onset 15–29: 16% 30–39: 44% 40–49: 28% ≥ 50: 12% Female: 19% White: 39% Hispanic: 21%	Social and community context (exposure)	Primary ocular lymphoma, squamous cell carcinoma of the conjunctiva, or primary ocular Kaposi sarcoma ‐ incidence (outcome)	This study found having HIV risk factors was associated with elevated incidence of various ocular cancers, squamous cell carcinoma of the conjunctiva, ocular Kaposi sarcoma, and primary ocular lymphoma.	Intramural Research Program of the National Cancer Institute, National Institutes of Health, and the Department of Health and Human Services
Inskip 2003	1974 to 1998	Cohort study	Patients diagnosed as having ocular melanoma between 1974 and 1998 identified in the SEER database (national)	3202	1992–1998 ‐ Age a diagnosis 0–49: 22% 50–64: 27% ≥ 65: 51% Female: 47% White: NR Hispanic: NR	Social and community context (exposure)	Ocular melanoma ‐ incidence (outcome)	This study found no rise in ocular melanoma over the study period but did find differences between races/ethnicities though they did not provide statistical analysis for these differences.	NR
Kim 2023	2006‐2019	Cohort study	Patients (≤ 65 years) diagnosed as having primary uveal melanomas between 2006 and 2013 and identified in the SEER database (national)	1765	Average age (SD): 51.8 (9.5) Female: 48% White: 95% Hispanic: NR	Economic stability; Education access and quality; Social and community context; Healthcare access and quality (exposure)	Uveal melanoma ‐ disease specific mortality (outcome)	This study found no evidence of associations between uveal melanoma‐specific mortality and insurance type, marriage status, or race.	None
Mahendraraj 2016	1973 to 2012	Cohort study	Patients diagnosed as having uveal melanoma between 1973 and 2012 and identified in the SEER database (national)	7516	Age < 50: 21% Age 50–79: 68% Age ≥ 80: 11% Female: 48% White: 95% Hispanic: 4%	Social and community context (exposure)	Uveal melanoma ‐ all‐cause mortality (outcome)	This study found that Hispanic patients had reduced odds of all‐cause mortality compared to non‐Hispanic patients and that males were at greater odds than females.	NR
McLaughlin 2005	1996 to 2000	Cohort study	Patients diagnosed as having noncutaneous melanoma between 1996 and 2000 and identified in the North American Association of Central Cancer Registries (national)	4885	Age 0–29: 3% Age 30–59: 38% Age 60+ : 59% Female: 49% White: 99% Hispanic: NR	Social and community context (exposure)	Ocular melanoma ‐ incidence (outcome)	This study found that the rate of ocular melanoma was more than eight times higher for Black males than White males and even higher for Black females compared to White females.	NR
Peters 2022	2011 to 2019	Retrospective chart review	Patients with choroidal or ciliochoroidal melanoma treated with iodine‐125 brachytherapy at the University of Miami Bascom Palmer Eye Institute between 2011 and 2019 (institutional)	258	Average age (SD): NR (NR) Female: 52% White: 90% Hispanic: 16%	Social and community context (exposure)	Uveal melanoma ‐ radiation side effects following brachytherapy (outcome)	This study found that race and ethnicity were not associated with the occurrence of radiation toxicity following brachytherapy for ocular melanoma.	None
Rajeshuni 2020	2004 to 2014	Cohort study	Individuals with uveal melanoma diagnosed between January 1, 2004, and December 31, 2014 and identified in the SEER 18 database (national)	4475	Age at diagnosis 0–55: 34% 56–68: 32% ≥ 69: 34% Female: 48% White: 92% Hispanic: NR	Economic stability; Education access and quality; Social and community context (exposure)	Uveal melanoma ‐ enucleation and radiation treatment (outcome) and disease specific mortality (outcome)	This study found that Non‐White patients and those in the lowest and middle SES tertile were more likely to undergo enucleation for ocular melanoma compared to non‐Hispanic White patients and those in the highest SES tertile, respectively.	The Stanford University 4 Medical Scholars Research Program, National Eye Institute, and Research to Prevent Blindness Inc.
Shields 2015	1970 to 2008	Retrospective chart review	All patients with uveal melanoma evaluated on the Ocular Oncology Service at Wills Eye Hospital between 25 August 1970 and 27 August 2008 (institutional)	8100	Average age (SD): 58 (NR) Female: 49% White: 98% Hispanic: 1%	Social and community context (exposure)	Uveal melanoma ‐ mortality (outcome) and metastasis (outcome)	This study found no statistical differences in metastases or death from uveal melanoma across races, although the strength of associations were large.	Wills Innovation Grant and the Eye Tumor Research Foundation
Shildkrot 2011	1996 to 2007	Retrospective chart review	All patients with the diagnosis of uveal melanoma seen at the University of Tennessee Health Science Center between June 1996 and August 2007, or at Ochsner Health System between April 1995 and May 2006 (regional)	635	Average age (SD): NR (NR) Female: NR White: 81% Hispanic: NR	Social and community context; Economic stability (exposure)	Uveal melanoma ‐ incidence (outcome)	This study found that zip codes with higher household values or higher average incomes were associated with an increased log‐rate of ocular melanoma.	Research to Prevent Blindness Inc. and the St. Giles Foundation
Tsai 2005	1973 to 2001	Cohort study	The age‐specific and age‐adjusted of cutaneous, ocular and visceral melanoma were compared in blacks, whites and other ethnic groups using data obtained from the SEER database for the years 1973–2001 (national)	NR	Average age (SD): NR (NR) Female: NR White: NR Hispanic: NR	Social and community context (exposure)	Ocular melanoma ‐ incidence (outcome)	This study found age‐adjusted incidence of ocular melanoma for White males (0.80), White females (0.62), Black males (0.08), Black females (0.04), Other males (0.08), and Other females (0.05).	NR
Williamson 2018	2007 to 2011	Cohort study	Patients recruited by research staff between June 1, 2007 and July 1 who were 18 or older, English speakers, and scheduled to receive diagnostic evaluation for an intraocular abnormality at the University of California, Los Angeles, Stein Eye Institute (state)	107	Average age (SD): 59 (13) Female: 46% White: 90% Hispanic: NR	Social and community context; Healthcare access and quality; Education access and quality (exposure)	Having at least one unmet social need (outcome)	This study found mixed evidence of associations between educational and social exposures and having unmet social needs.	National Institute of Mental Health, the Jonsson Comprehensive Cancer Center Foundation, George E. and Ruth Moss Trust, and Research to Prevent Blindness Inc.
Yu 2003	1973 to 1999	Cohort study	Patients diagnosed as having conjunctival melanoma from 1973 to 1999 and identified in the SEER database (national)	206	Age < 40: 13% 40–59: 24% ≥ 60: 63% Female: 41% White: 91% Hispanic: NR	Social and community context (exposure)	Conjunctival melanoma ‐ incidence (outcome)	This study looked at cumulative incidence of conjunctival melanoma.	NR
Zhang 2022	2004 to 2015	Cohort study	Patients diagnosed as having ocular surface squamous neoplasia from 2004 to 2015 identified in the National Cancer Database (national)	2402	Median age: 69 Female: 29% White: 95% Hispanic: NR	Social and community context; Education access and quality; Economic stability; Healthcare access and quality (exposure)	Ocular surface squamous neoplasia ‐ mortality (outcome)	This study found positive associations between race and insurance type with hazard of mortality due to ocular surface squamous neoplasia, but no associations with zip code or income levels.	None

Abbreviations: Inc, incorporated; NR, not reported; SD, standard deviation; SEER, Surveillance, Epidemiology, and End Results; US, United States.

Across the 21 included studies, a wide variety of exposures representing four SDOH domains were investigated, with social and community context being the most frequently examined, primarily through measures of race and ethnicity. Other commonly assessed exposures included economic stability factors (e.g., income or socioeconomic status), neighborhood and built environment conditions (e.g., living in urban vs. rural settings), healthcare access indicators (e.g., insurance type), and educational attainment levels. Outcomes primarily focused on survival and mortality, treatment type, and the incidence of uveal melanoma. None of the studies covered the Neighborhood and Built Environment domain.

### 3.2 Risk of Bias of Included Studies

**FIGURE 2:**
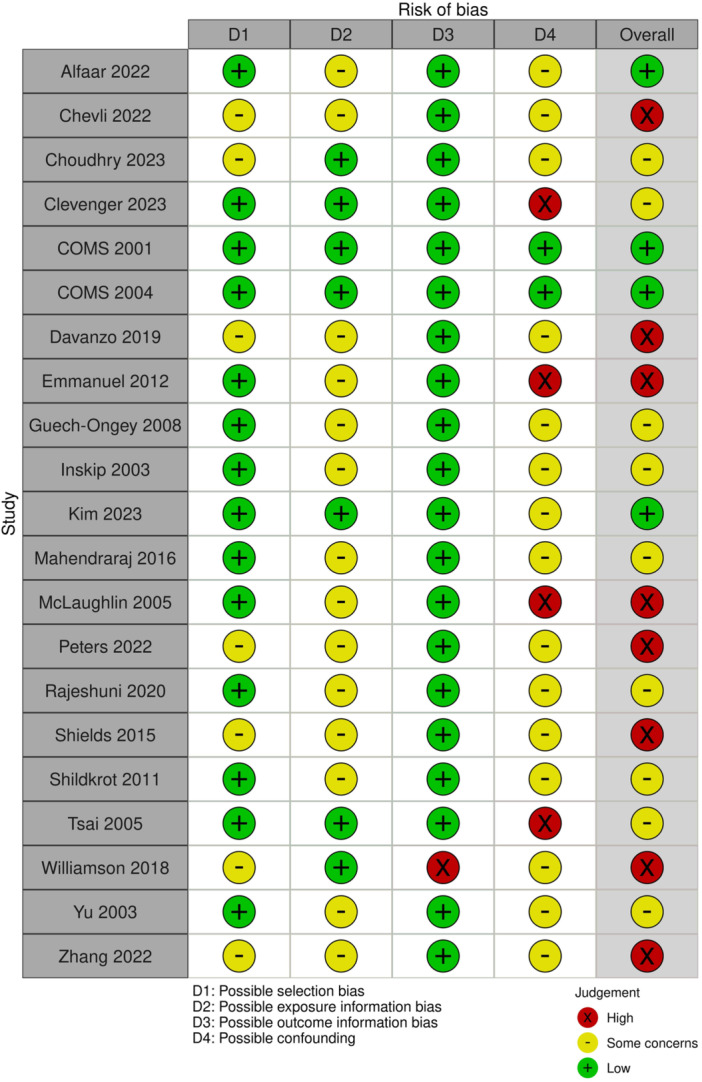
Risk of bias stoplight plot.

Of the 21 included studies, we judged 8 (38%) to have a high risk of bias [12, 13, 14, 15, 16, 17, 18, 19, 20], 9 (43%) to have a moderate risk of bias [21, 22, 23, 24, 25, 26, 27, 28, 29], and 4 (19%) to have a low risk of bias [30, 31, 32] (Table S1). Figure 2 displays the risk of bias assessments for individual studies. Overall, the primary areas of concern regarding bias were participant selection, exposure information bias (i.e., misclassification), and potential confounding of results due to incomplete control of confounding variables in analyses and/or study design.

### 3.3 Associations

**FIGURE 3:**
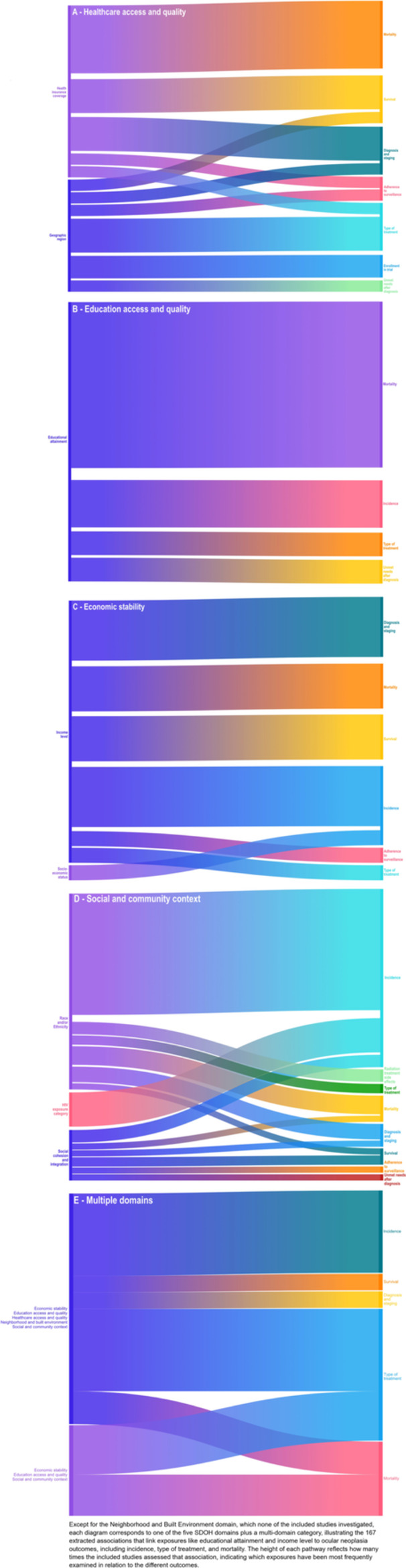
(A–E) Associations between social determinant domains and adult ocular neoplasia outcomes. Except for the Neighborhood and Built Environment domain, which none of the included studies investigated, each diagram corresponds to one of the five SDOH domains plus a multi‐domain category, illustrating the 167 extracted associations that link exposures like educational attainment and income level to ocular neoplasia outcomes, including incidence, type of treatment, and mortality. The height of each pathway reflects how many times the included studies assessed that association, indicating which exposures have been most frequently examined in relation to the different outcomes.

Across the 21 studies, we found 167 associations between SDOH and ocular cancer outcomes. Figure 3 presents five Sankey plots corresponding to four SDOH domains plus a multi‐domain category (except Neighborhood and Built Environment). In the plots, the height of each pathway reflects how many times the included studies assessed that association, indicating which exposures have been most frequently examined across the different outcomes. Most domains were assessed using only 1 or 2 exposure categories, and these exposures often mapped to multiple outcomes. For example, educational attainment was assessed for all four outcome categories of interest (mortality, cancer incidence, type of treatment received, and unmet needs after diagnosis). Studies could assess multiple SDOH across multiple domains.

We present narrative summaries of the evidence according to the social determinant domain and organized by outcome, with the most commonly studied cancers presented first. Table S2 presents all associations, estimates, domains, and directions of effect for included studies and allows filtering by outcome for further exploration. Table 2 provides a summary of association directionality (favorable, unfavorable, null, or not applicable) by SDOH domain.

**TABLE 2:** Directionality of associations between SDOH and ocular‐cancer outcomes by SDOH domain.

SDOH domain	Total associations	Directionality of associations (*n* = 167)			
		Unfavorable	Favorable	Null	Not applicable
Healthcare access and quality	24	12	7	2	3
Education access and quality	11	9	2	0	0
Ecomnomic stability	17	9	5	1	2
Social and community context	85	31	24	0	30
Multiple domains	30	14	4	0	12

#### 3.3.1 Healthcare Access and Quality

Seven studies examined 24 associations with four different ocular neoplasms, or groups of neoplasms, within the healthcare access and quality domain, spanning facility academic status or experience level, travel distance or geographic proximity to care, and insurance status. Twelve of the 24 associations (50%) were classified as unfavorable (associated with worse ocular‑cancer outcomes), 7 (29%) were favorable (related to improved outcomes), 2 (8%) were “null” (no clear relationship), and 3 (13%) did not have directionality (“NA”).

*Uveal melanoma*: Chevli 2022 (high risk of bias) reported that among patients with small/medium uveal melanoma tumors, treatment at an experienced center or even traveling over 60 miles for care were both associated with a lower risk of enucleation (RR = 0.45 [0.40, 0.50] and 0.63 [0.56, 0.71], respectively). In contrast, receiving care at an academic center or having no insurance corresponded to a higher risk of requiring enucleation, with uninsured patients experiencing more than double the risk (RR = 2.23 [1.77, 2.80]) [12]. This association may reflect selection bias, as patients with more advanced or complex disease may be at higher risk for enucleation to begin with, and are more likely to be referred to academic centers. Williamson 2018 (high risk of bias) found that higher baseline instrumental support scores are associated with fewer self‑reported unmet needs 3 weeks post uveal melanoma diagnosis (β = –0.20 [–0.40, –0.10]) [19]. Kim 2023 (low risk of bias) observed lower hazard of death among Medicaid‑insured uveal melanoma patients compared to those with non‐Medicaid insurance (aHR = 0.75 [0.39, 1.45]) [33].

*Uveal/conjunctival melanoma and retinoblastoma*: Choudhry 2023 (moderate risk of bias) reported that insurance status, rather than travel distance, was the key factor associated with disease severity. Patients on Medicaid or without insurance had higher odds of presenting with advanced‐stage tumors (aOR = 1.88 [1.32, 2.66] and 1.74 [1.16, 2.60], respectively), whereas longer travel distance had no impact on 5‐year survival [21]. Similarly, Zhang 2022 (high risk of bias) found that ocular surface squamous neoplasia patients with government insurance or no insurance experienced higher mortality compared to those with private coverage [18].

*Choroidal melanomas*: The COMS 2001 trial (low risk of bias) showed that living in the same state as the treatment center increased enrollment in clinical trials for both large (aOR = 2.20 [1.62, 3.00]) and medium choroidal melanomas (aOR = 1.38 [1.16, 1.64]) [31].

#### 3.3.2 Education Access and Quality

Five studies examined 11 associations between three different ocular neoplasms and both individual and county‐level education measures of the education access and quality domain. Nine of the 11 associations (81.8%) were categorized as unfavorable and 2 (18.2%) were favorable.

*Uveal melanoma*: Chevli 2022 (high risk of bias) found that patients with low‑education had a higher risk of enucleation for small/medium uveal‑melanoma tumors (RR = 1.15 [1.03, 1.29]) [12]. Similarly, Clevenger 2023 (moderate risk of bias) noted that uveal melanoma incidence was elevated in populations with fewer college‐educated residents, with the least‐educated counties experiencing higher incidence than the most‐educated (RR = 1.18 [1.01, 1.37] for lowest vs. highest education quartile) [22].

*Choroidal melanoma*: Within the COMS 2004 study (low risk of bias), participants whose highest attainment was a high‑school diploma had greater 10‑year mortality from metastatic choroidal melanoma when they received pre‐enucleation radiation compared with enucleation alone (RR = 1.40 [1.08, 1.83]), and a similar, though less precise, increased risk at 5 years (RR = 1.33 [0.98, 1.81]). Education beyond high school was associated with a 5 and 10‐year survival benefit (RR = 0.78 [0.54, 1.13] and RR = 0.60 [0.38, 0.94], respectively) [32].

*Ocular surface squamous neoplasia*: Zhang 2022 (high risk of bias) reported that living in areas with a higher percentage of adults lacking a high‑school diploma (greater than 21%) might be associated with higher mortality from ocular surface squamous neoplasia (HR = 1.10 [0.83, 1.46]) [18].

#### 3.3.3 Economic Stability

Six studies investigated 17 associations within the economic‑stability domain and three different ocular neoplasms, or groups of neoplasms, focusing on individual or county‑level income and housing value. Nine associations (52.9%) were unfavorable, 5 (29.4%) were favorable, 1 (5.9%) was null, and 2 (11.8%) were not assigned a direction (NA).

*Uveal melanoma*: Chevli 2022 (high risk of bias) reported that patients in the lowest income category had a lower risk of enucleation for small/medium uveal melanoma tumors (RR = 0.73 [0.65, 0.83]) [12]. Clevenger 2023 (moderate risk of bias) found higher uveal melanoma incidence in counties at the highest median‑income stratum (> $80 K; RR = 1.54 [1.41, 1.69]) and in the third‑highest stratum ($57K–$68K; RR = 1.25 [1.06–1.48]) compared with the lowest (<$44 K). The second‑lowest stratum ($44K–$56K) showed no clear association (RR = 0.95 [0.84, 1.07]) [22]. Davanzo 2019 (high risk of bias) found no evidence of difference in uveal melanoma metastatic risk across income categories (*p* = 0.41) [13]. Shildkrot 2011 (moderate risk of bias) noted a positive association between average housing value and uveal melanoma incidence (β = 0.38, SE = 0.12) [27]. These findings may reflect detection bias, as higher‐income individuals may have better access to care and thus are more likely to be diagnosed.

*Uveal/conjunctival melanoma and retinoblastoma*: In contrast to Chevli 2022, who only looked at uveal melanoma, Choudhry 2023 (moderate risk of bias) observed that, compared with the lowest income quartile (<$46 K), being in the second income quartile ($46K–$58K) was associated with reduced odds of advanced tumor presentation (aOR = 0.72 [0.55, 0.94]), but had unchanged 5‐year survival (HR = 1.00 [0.83–1.20]). Both the third ($58K–$74K) and fourth (>$74K) income quartiles had both reduced odds of advanced tumor presentation and improved 5‐year survival compared with the lowest quartile [21] (Table S2).

*Ocular surface squamous neoplasia*: Zhang 2022 (high risk of bias) examined ocular surface squamous neoplasia mortality across four income brackets. Patients with high incomes ($48K–$53K) had roughly the same morality as their highest income group counterparts (>$63K; HR = 0.99 [0.79, 1.25]), whereas low (<$38K) and moderate ($38K–$48K) incomes indicated modest increased mortality (HR = 1.07 [0.80, 1.42] and HR = 1.13 [0.92, 1.39], respectively) compared with those in the highest income group [18].

#### 3.3.4 Social and Community Context

Eighteen studies examined 85 associations with various measures of the social and community context domain including race and ethnicity, marital status, sex, and social support. Thirty‑one of the 85 associations (36.5%) were unfavorable, 24 (28.2%) were favorable, none were null, and 30 (35.3%) lacked directional assignment (NA).

*Uveal‑melanoma*: Clevenger 2023 (moderate risk of bias) found increased uveal melanoma incidence in countries with lower proportions of non‐White residents (RR = 1.17–1.28) [22]. Rajeshuni 2020 (moderate risk of bias) observed that non‐White patients had higher odds of enucleation than non‐Hispanic White (aOR = 1.45 [1.12, 1.88]) [26]. Alfaar 2022 (low risk of bias) found that married uveal‑melanoma patients had an 18% lower 10‑year cancer‑specific mortality than divorced patients (aHR = 0.82 [0.69, 0.98]), while widowed status showed null assositiaon (aHR = 1.02 [0.82, 1.26]) [30]. Shildkrot 2011 (moderate risk of bias) showed that a higher number of persons‑per‑household predicted lower uveal melanoma incidence (β = –0.29, SE 0.08), but an interaction with population size reversed the relationship (β = 0.14, SE 0.07) [27]. Williamson 2018, (high risk of bias) found that higher baseline emotional support was possibly associated with fewer unmet supportive‑care needs 3 weeks after diagnosis (β = –0.10 [–0.30, 0.20]) [19], whereas Davanzo 2019 (high risk of bias) reported no evidence of difference in surveillance adherence or metastasis risk between support groups [13]. Mahendraraj 2016 (moderate risk of bias) observed that males with uveal melanoma had slightly higher mortality odds than females (OR = 1.10 [1.00, 1.30]) [25].

*Uveal/conjunctival melanoma and retinoblastoma*: Choudhry 2023 (moderate risk of bias) found that identifying as Black might be associated with a greater odds of advanced tumor stage at presentation (aOR = 1.27 [0.71, 2.26]) and, paradoxically, an improved 5‑year survival (HR = 0.80 [0.46, 1.40]) for patients diagnosed with uveal melanoma, conjunctival melanoma, or retinoblastoma [21].

*Ocular Kaposi sarcoma, ocular lymphoma, and conjunctival squamous‑cell carcinoma*: There are relatively few studies examining the specific associations between ocular Kaposi sarcoma, ocular lymphoma, and conjunctival squamous‐cell carcinoma and SDOH [18, 23], though the associations between HIV/AIDS and SDOH is well documented [34, 35]. In an HIV cohort, Guech‑Ongey 2008 (moderate risk of bias), found that the incidence of ocular Kaposi sarcoma, ocular lymphoma, and conjunctival squamous‑cell carcinoma was higher among Black and Hispanic persons than expected, with standardized incidence ratios ranging from 10.7 to 123.0 [23].

*Ocular‑surface squamous‑neoplasia*: Similar to Guech‐Ongey 2008, Zhang 2022 (high risk of bias), found that patients who identified as Black had a greater hazard of mortality from ocular surface squamous neoplasia than White patients (HR = 1.66 [1.37, 2.01]) [18].

#### 3.3.5 Multiple Domains

Seven studies evaluated 30 multi‑domain associations between uveal melanoma and measures such as socioeconomic‑status indices, geographic region, and rurality classifications; 14 associations (46.7%) were unfavorable, 4 (13.3%) were favorable, and 12 (40.0%) lacked directional assignment (NA).

*Uveal melanoma*: As with single‑domain findings, being part of the lower socioeconomic status, or related disadvantage, generally corresponded to more advanced disease at diagnosis, higher odds of enucleation, and greater uveal melanoma incidence or mortality [12, 21, 22, 26, 27, 30, 33].

## 4 Discussion

Our systematic review identified 21 US studies reporting 167 unique associations between SDOH measures and outcomes for uveal melanoma, conjunctival squamous cell carcinoma, ocular lymphoma, and ocular Kaposi sarcoma. Although fewer than half of the estimates found an association, the direction of the association was predominantly unfavorable for individuals experiencing economic hardship, limited educational opportunities, non‐White race or ethnicity, non‐private insurance, or care delivered outside high‐volume centers. In contrast, several studies indicated that higher income, private insurance, and treatment at experienced facilities were generally, though not uniformly, linked to an earlier stage at presentation, a lower likelihood of enucleation, and improved survival.

Our synthesis aligns with the key themes highlighted in a recent narrative review of health outcomes in ocular oncology [36]. Across both analyses, minority racial and ethnic groups, patients in the lowest income strata, and those with public or no insurance were more likely to undergo primary enucleation, although survival findings are mixed. Our review extends those observations by mapping the SDOH framework to studies of ocular cancers and demonstrating that the same social gradients operate across malignancies that vary by patient age, disease etiology, and treatment.

Several findings in our review differed from conventional expectations, namely that “worse” exposures would be linked to “worse” outcomes. For example, patients treated at academic centers had higher risk of enucleation [12], while lower‐income patients with small or medium tumors had a lower risk [12]. Similarly, uveal melanoma incidence was greater in higher‐income areas [22]. These paradoxical findings likely reflect residual confounding or biases. Interpretation of these findings must account for several forms of bias inherent to observational and registry‐based studies of SDOH. Confounding by indication is particularly relevant for treatment outcomes, as decisions such as enucleation are driven by tumor characteristics and prior treatment response that may not be fully captured in secondary datasets. Referral bias may further concentrate patients with advanced or refractory disease at academic and high‐volume centers, inflating observed rates of enucleation independent of care quality [37]. Detection bias likely contributes to higher reported uveal melanoma incidence in wealthier or better‐insured populations due to greater access to routine eye care and diagnostic evaluation [38]. Finally, stage migration may occur in specialized centers with more detailed diagnostic workups, leading to reclassification of disease severity without a true change in underlying disease state [39]. For example, sicker patients are likely treated at academic centers, which manifests as the patients at those centers appearing to have a higher incidence of enucleation compared with those treated elsewhere. In uveal melanoma, pattern may reflect referral of patients whose tumors fail initial globe‐sparing therapies and subsequently require enucleation at tertiary centers [26]. Notably, stage at presentation does not appear to differ significantly by socioeconomic status, suggesting that differences in incidence may reflect variation in detection or case ascertainment rather than true disparities in disease burden [26].

Additionally, as social constructs, factors like race, income, or insurance status are used as proxies rather than precise variables, and interpreting them requires caution. Ultimately, SDOH are inherently interlinked and rarely act upon health in isolation. Their interdependence complicates our ability to isolate the effect or effects of any single factor. For example, the impact of race or ethnicity on ocular cancer outcomes may differ by insurance status, geographic proximity to care, or rural versus urban residence, while the effect of distance to care may be modified by insurance coverage or neighborhood resources. Most included studies examined single‐domain associations and were not designed to assess such interactions. Incorporating intersectional frameworks (e.g., stratified analysis, using interaction terms, multilevel modeling) may help explain heterogeneous or paradoxical findings and improve the targeting of equity‐focused interventions.

Our findings underscore that multi‐domain interventions (e.g., a combination of expanding insurance coverage, subsidizing travel to high‐volume centers, and strengthening community eye health infrastructure) are required to mitigate delays in diagnosis and optimize treatment selection. Investigators could also examine how multi‐domain identities (e.g., race and rural residence) modify risk and evaluate whether policy changes (e.g., Medicaid expansion) translate into measurable ocular‑cancer benefits. For example, the impact of insurance expansion on stage at diagnosis, treatment selection, and survival could be assessed using difference‐in‐differences designs comparing outcomes before and after Medicaid expansion across states. Similarly, before–after or interrupted time‐series analyses could be used to evaluate referral pathways or regionalization initiatives aimed at increasing access to high‐volume ocular oncology centers. Embedding such evaluation frameworks into policy and health‐system changes would strengthen causal inference and help guide scalable implementation across health systems.

A strength of evidence is the standardized ascertainment of cancer outcomes through registry‐based data, which was used by over half of the included studies, thereby enhancing comparability across studies. We also applied uniform rules to classify directionality. However, heterogeneity in exposure definitions, confounder adjustment, and analytic approaches prohibited the use of quantitative meta‐analysis. We judged nearly 40% of studies to have a high risk of bias, primarily due to possible exposure misclassification and uncontrolled confounding. Finally, few studies reported outcomes beyond 5 years, which may not capture late metastasis, and the impact of SDOH may widen over longer periods.

## 5 Conclusion

From the probability of an early diagnosis to the treatments offered and the chances of survival, SDOH are associated with the entire ocular‑cancer experience. In US populations, lower socioeconomic status, limited insurance coverage, minority racial and ethnic identity, and residence distance from specialized care were consistently associated with worse outcomes for uveal melanoma and other rare ocular tumors. Addressing these gaps will require coordinated clinical, community and policy interventions that span multiple SDOH domains.

## Author Contributions

**Daniel Shaughnessy:** investigation, writing – original draft, methodology, validation, visualization, writing – review and editing, data curation, formal analysis. **Vijay Joshi:** investigation, writing – original draft, methodology, validation, visualization, writing – review and editing, formal analysis. **Natalia Dellavalle:** investigation, writing – original draft, writing – review and editing, validation. **Louis Leslie:** investigation, writing – original draft, visualization, methodology, validation, writing – review and editing. **Michael Edwards:** writing – review and editing, writing – original draft, investigation, methodology. **Timothy Waxweiler:** investigation, writing – original draft, writing – review and editing. **Tianjing Li:** funding acquisition, conceptualization, investigation, methodology, writing – review and editing, writing – original draft, project administration. **Riaz Qureshi:** conceptualization, investigation, writing – original draft, methodology, validation, visualization, writing – review and editing, project administration.

## Conflicts of Interest

The authors declare no conflicts of interest.

## Supporting information


**Appendix A:** Detailed methods for systematic review of social determinants of health and ocular neoplasia.

**Appendix B:** Combined SRDR+ and qualtrics data extraction forms.

**Table S1:** Risk of bias assessments for the included studies.

**Table S2:** Associations identified by included studies.

## Data Availability

The data that support the findings of this study are available in the supporting information of this article.
