# Supplementary material for: Associations of Social and Demographic Factors on the Outcomes of Ocular Melanoma and Other Adult Ocular Neoplasms in the United States: A Systematic Review
Source: Cochrane Evid Synth Methods. 2026 Mar 10;4(2):e70075. doi: 10.1002/cesm.70075 (PMC12977123; doi:10.1002/cesm.70075)
Supplement: Supplementary file 1 — Appendix A: Detailed methods for systematic review of social determinants of health and ocular neoplasia. [file CESM-4-e70075-s003.DOC]

**APPENDIX A – Detailed methods for systematic review of social determinants of health and ocular neoplasia**

We performed this review within a suite of systematic reviews being undertaken by Cochrane Eyes and Vision US Project (CEV@US) examining various aspects of eye health and their relation to the SDOH.^1^ In this review, we focused on ocular neoplasia and followed a protocol that is published in full on Open Science Framework.^2^ No ammendments were made to the protocol. There are separate reviews that examine the relationship between SDOH and other relevant eye and vision topics and conditions such as dry eye, pediatric vision screening, telehealth, etc.^1-5^ A review by Korn et al. looked at the relationships between breast, cervical, colorectal, and lung cancer screening and SDOH, which provided a helpful model to set up our own literature search and overall approach in addition to the standard protocol established for the suite of reviews.^6^

**2.1 Eligibility criteria**

Study Design: Eligible studies were primary studies that examined the relationship between SDOH and ocular neoplasia, utilized an observational or interventional design, were reported in English, and had findings published after 2000. Both quantitative and qualitative studies were eligible. We excluded studies revealing findings of biological and mechanistic causes of diseases. As an example, we would have excluded a study looking at genetic profiling of ocular neoplasms. We also excluded studies that were conducted exclusively in one subpopulation (restriction) without a comparison group. For example, we would have excluded a study that described ocular neoplasia diagnosed in a single hospital but did not compare this group with another group. Finally, we excluded studies that assessed the cost-effectiveness of different interventions.

Population: We included studies in populations with ocular neoplasia(e.g., ocular melanoma). We excluded studies in populations of retinoblastoma alone, non-ocular neoplasias (e.g., cutaneous melanoma) that do not involve the eye or surrounding structures, as well as central nervous system tumors (e.g., pituitary adenomas, craniopharyngiomas, optic nerve sheath meningiomas) and skull base tumors (e.g., meningiomas, chordomas, chondrosarcomas, nasopharynx cancers) that can affect vision by compressing optic nerves, chiasm, or tracts. We included participants from any study setting; for example, any relevant population-based, hospital/clinic-based, community-based, and school-based study was eligible. As our focus is on the US context because social determinants are highly culturally dependent, we only included studies conducted on (or including) US populations.

Exposure/Intervention(s): We included studies that focused on at least one SDOH within the five domains defined by the Healthy People 2030 framework^7^, as well as studies that investigated various indices as composite measures of SDOH (e.g., Yost index^8^). Associations may be multi-directional – thus we included studies when SDOH is used either as exposure or outcome. We included intervention studies that attempt to ameliorate SDOH. For example, we would have included a study that examined whether implementing community-based care in underserved areas influenced the stage at diagnosis of ocular neoplasms. We also included studies that examined whether SDOHs are barriers, facilitators, and/or moderators of ocular neoplasia diagnosis and treatment effectiveness. For example, we would have included a study that looked at the relationship between food or housing insecurity and ocular melanoma treatment adherence. We excluded studies that included SDOH only as demographic or control variables. For example, if a study included race and ethnicity in a regression model, but the primary goal was not to examine the relationship between SDOH variables and ocular neoplasia, this study would have been excluded.

Outcome: We did not exclude studies based on specific outcomes reported. As mentioned previously, the association could be bi-directional: we included studies relating SDOH to ocular neoplasia (e.g., the effect of socioeconomic disparities on cancer survival) as well as studies relating ocular neoplasia to SDOH (e.g., whether cancer diagnosis affects an individual's sense of social isolation or cohesion).

**2.2 Selection of studies**

For this review, we identified eligible studies from a master database on social determinants of ocular health that was developed and maintained by CEV@US. For the master database, a comprehensive literature search of Ovid MEDLINE, Embase.com, and Web of Science Core Collection was conducted on November 23, 2024. Sub-appendix A presents the full search strategy. Researchers from CEV@US pilot tested title and abstract screening and the full-text screening and tagging procedure before the formal review process began in Covidence. Two individuals worked individually in pairs to screen against the eligibility criteria for each title and abstract and full-text reports when deemed relevant. At the full text reviewing stage, the pair of researchers tagged each eligible record by eye condition and topic, SDOH domain, report type, study design, and age group. At both stages, we resolved discrepancies regarding eligibility for the master database through discussion and/or consultation with a third individual.

We selected all full-text reports tagged as 'ocular cancer' during the primary full-text screening stage for the master database. We included all types of ocular neoplasms (e.g., retinoblastoma, uveal melanoma, etc.) and neoplasms affecting the area around the eye or orbit (e.g., periocular cutaneous basal and squamous cell carcinoma). We also included the studies that grouped ocular and non-ocular neoplasms for further evaluation. Given the richness of associations we found, we focused this review solely on non-retinoblastoma ocular cancers and noted reports related to retinoblastoma for later use. For reviews and non-primary studies that satisfied the eligibility criteria for this review, we searched the reference lists for potentially eligible primary studies.

Two people worked independently to screen the studies selected addressing non-retinoblastoma ocular neoplasias for inclusion for this review. We documented reasons for exclusion. We generated a study flow diagram that describes the identification of studies. We resolved disagreements through discussion.

**2.3 Data extraction and risk of bias assessment**

We piloted a data extraction form using Systematic Review Data Repository (SRDR+) and Qualtrics. For each included study, one author extracted data and assessed the potential risk of bias, which a second author verified. We resolved discrepancies through discussion or adjudication by a third reviewer.

We extracted the following items related to the: (a) study (title, journal, author(s), year of publication, study objectives, design, sampling method, sample size participants in analysis, dates of follow-up, follow-up period as reported, and other information regarding where and how the participants were recruited), (b) population (eye condition and severity, age, sex, gender, race, ethnicity, and other demographic characteristics), (c) exposure (SDOH mapped to five Healthy People 2030 domains [economic stability, neighborhood and built environment, healthcare access and quality, education access and quality, and social and community context]^7^, constructs^1^, measures, and level of measurement, details of the interventions and factors relevant to implementation), (d) outcome (incidence, progression, and severity of neoplasia; visual impairment definition and frequency; treatment adherence; generic and vision-related quality of life; SDOH construct or variable (when used as outcomes)), (e) association (effect estimates (e.g., odds ratio) and associated measures of precision, and key findings and conclusions), and (f) risk of bias (see below).

Given the heterogenous set of study designs expected, we assessed the risk of bias using domains adapted from the Newcastle-Ottawa Scale.^9^ The Cochrane Eyes and Vision group has used this assessment for other systematic reviews in the broader social determinant of health project as it allows for a general measure of the potential for bias that is applicable to multiple different study designs and settings. The assessment includes five domain judgments and justifications, including: (1) Representativeness of the sample (in cross-sectional studies) or whether the study sample was free of selection bias (in other study designs), (2) Risk of information bias in the measurement of exposures, (3) Risk of information bias in the measurement of outcomes, (4) Risk of information bias due to missing data, and (5) Risk of bias due to confounding.

**2.4 Data synthesis**

We synthesized results using narratives, tables, and figures. We grouped the results by SDOH domain, population (e.g., ages, setting), and study design (e.g., observational, interventional). We anticipated a heterogenous set of studies that would not be amenable to meta-analysis. We used alternative methods such as Harvest plots and Sankey figures to guide a qualitative synthesis following the guidance described in Chapter 12 of the Cochrane Handbook.^10^

To help our qualitative synthesis, we classified each extracted association based on its statistical significance and direction of effect. Association directionality was classified as follows: favorable if "worse" exposure, compared with "better" exposure, was associated with improved outcomes (e.g., reduced incidence); unfavorable if "worse" exposure was linked to worse outcomes (e.g., increased mortality or extreme treatments like enucleation); and null if no clear relationship was observed. For instance, if a study found that lower income levels were associated with increased mortality compared with higher income levels, we classified this as unfavorable. This classification logic was applied consistently across all exposures, including distance to agriculture (closer vs. further away), insurance type (public or none vs. private), chemical exposure (any vs. none), and race/ethnicity (any minority group vs. majority group). In some cases, the directionality of the exposure-outcome relationship could not be determined (e.g., lack of a clearly defined reference group or multi-group tests for homogeneity that did not yield a single comparison group), and these associations were labeled "NA" to avoid erroneous assignment of favorable or unfavorable status. For example, an analysis that evaluated poverty level by race and ethnicity in four groups (White non-Hispanic, White Hispanic, Black, and Others) via a Mantel-Haenszel test for homogeneity that used no definitive reference category was classified as "NA." For the studies that only reported P-values and did not report effect estimates for their associations, we classified the direction as "Not applicable".

We identified a variety of outcome types, including cancer incidence, effects of treatment and care that patients received, and mortality/survival. We classified the outcomes into one of the following general categories to organize our synthesis: adherence to cancer surveillance, cancer-specific or all-cause mortality, cancer survival, developmental delay/school difficulties, diagnosis and staging of cancer, cancer incidence, radiation treatment side effects, and type of ocular cancer treatment. We organized our results to present associations for the outcomes in the following order, where reported: mortality or survival, type of treatment, having advanced disease at diagnosis, and cancer incidence. Because no meta‑analysis was undertaken, sensitivity analyses of pooled effect estimates were not applicable. Additionally, per the protocol, we did not conduct assesments for reporting bias or certainty of evidence.^2^

**References**

1. Li T, Abraham, A., Collins, M., Ehrlich, J.R., Elam, A., Lamoureux E., Piper C., Summers A. Social determinants of eye health in the United States: a systematic review protocol. 2023;doi:10.17605/OSF.IO/M8B4Q

2. Joshi V* SD, Dellavalle N, Leslie L, Edwards M, Luna-Fineman S, Waxweiler T, Hawkins B, Li T, Qureshi R. . Social determinants of ocular neoplasia in the United States: a systematic review protocol. *Open Science Framework*. February 6, 2024 2024;doi:<https://doi.org/10.17605/OSF.IO/6JSPZ>

3. Liu SH, Shaughnessy D, Leslie L, et al. Social Determinants of Dry Eye in the United States: A Systematic Review. *Am J Ophthalmol*. May 2024;261:36-53. doi:10.1016/j.ajo.2024.01.015

4. John Gorham AdAC, Angell Shi, Louis Leslie, Anne Lynch, Nicholas Quan, Tianjing Li. Social determinants of retinopathy of prematurity in the United States: a systematic review protocol. Protocol. *Open Science Framework*. January 22, 2024 2024;doi:<https://doi.org/10.17605/OSF.IO/GEJT7>

5. Choo A. LL, Tzang C., Liu S., Li T., Kuo I. Social determinants of cataract surgery in the United States: a systematic review protocol. *Open Science Framework*. 2024;doi:10.17605/OSF.IO/2X3WK

6. Korn AR, Walsh-Bailey C, Correa-Mendez M, et al. Social determinants of health and US cancer screening interventions: A systematic review. *CA Cancer J Clin*. Sep-Oct 2023;73(5):461-479. doi:10.3322/caac.21801

7. Social determinants of health. U.S. Department of Health and Human Services. Accessed September 22, 2023. <https://health.gov/healthypeople/priority-areas/social-determinants-health>

8. Yost K, Perkins C, Cohen R, Morris C, Wright W. Socioeconomic status and breast cancer incidence in California for different race/ethnic groups. *Cancer Causes Control*. Oct 2001;12(8):703-11. doi:10.1023/a:1011240019516

9. GA Wells BS, D O'Connell, J Peterson, V Welch, M Losos, P Tugwell,. The Newcastle-Ottawa Scale (NOS) for assessing the quality of nonrandomised studies in meta-analyses. Accessed July 11, 2022. <https://www.ohri.ca/programs/clinical_epidemiology/oxford.asp>

10. McKenzie JE BS. Chapter 12: Synthesizing and presenting findings using other methods [last updated October 2019]. In: Higgins JPT TJ, Chandler J, Cumpston M, Li T, Page MJ, Welch VA, ed. *Cochrane Handbook for Systematic Reviews of Interventions version 65*. Cochrane; 2024:chap 12.

**Sub-appendix 1**. Search strategies

| Database(s): Ovid MEDLINE(R) ALL 1946 to November 23, 2024 | |
| --- | --- |
| **#** | **Searches** |
| 1 | exp "Eye Diseases"/ |
| 2 | (((refractive or refraction) adj2 (error* or disorder*)) or "wavefront aberration*" or Amblyopia* or "lazy eye" or amtropia* or Aniseikonia or Anisometropia* or astigmatism* or (corneal adj3 abberation*) or Hyperopia or Presbyopia or ((eye or vitreous or "anterior chamber" or choroid or retinal or conjunctival or ocular) adj2 h?emorrhage) or Hyphema* or (aqueous adj2 "outflow obstruction*") or Asthenopia or "Balint* Syndrome" or Hemianopia* or Hemianopsia* or Pseudoaphakia* or "capsule opacification*" or (Chorioretinal adj2 (Disorder* or disease*)) or Chorioretinitis or Chorioretinitides or "Choroid Disease*" or Choroidal or Choroiditis or Choroiditides or Chromatopsia or Epiphora* or Diplopia* or Polyopsia* or Ophthalmia* or Episcleritis or Scleritis or (scleral adj2 disease*) or ((equatorial or scleral or anterior or posticum) adj2 staphyloma) or strabismus or exotropia or Esotropia* or Esodeviation* or "orbital disease*" or enophthalmos or Exophthalmos or Proptos?s or ophthalmopath* or Ophthalmoplegia* or "eye muscle paralysis" or (Fuchs adj3 (dystrophy or atrophy)) or Nystagmus or Oculopath* or (optic adj3 (atrophy or disease* or disorder* or glioma* or injur* or neuritis or papillitis)) or Papilledema* or "optic disk edema*" or "optic oedema*" or "choked disk*" or Photalgia or Photophobia* or Photopsia* or "Retrobulbar Neuritis" or scotoma* or (vitreous adj2 (detachment* or strand* or vein* or prolapse* or syneresis))).tw,kf. |
| 3 | (Anophthalmos or Anophthalmia* or (eye adj2 abnormalit*) or Vitreoretinopath* or Hydrophthalmos or Microphthalmos or "retinal dysplasia*").tw,kf. |
| 4 | ("dry eye" or "dry eyes" or (lacrimal adj3 (disorder* or disease* or obstruction*)) or ((decreased or abnormal) adj3 lacrimation) or "Meibomian Gland Dysfunction*" or "MG Dysfunction*" or Meibomianitis or Meibomianitides or Xerophthalmia*).tw,kf. |
| 5 | ((visual adj3 (loss or agnosia* or defect* or difficult* or disorder* or disturbance* or constriction* or impair*)) or ((loss or abnormal or blurred or decreased or defect* or deficienc* or disorder* or dim or difficulty or diminished or disturbed or disturbance or hazy or impair* or interference or reduced or weak or partial or subnormal or tunnel or problem) adj2 vision) or Hypermetropia or Farsighted* or "far sighted" or Myopia or nearsighted* or "near sighted" or Blindness or "legal* blind*" or "Delayed Visual Maturation" or "difficulty seeing" or "low vision" or "impaired visual acuity" or "partially sighted" or ((impair* or partial) adj2 sight) or (sunken adj2 (eye or eyes or eyelid)) or Symblepharon).tw,kf. |
| 6 | (eyestrain or "eye fatigue" or "eye strain" or "visual fatigue" or "eye redness").ti,ab. |
| 7 | ((eye adj2 (injur* or Burn* or "foreign bodies" or "foreign body" or discharge*)) or "traumatic hyphema*" or (corneal adj2 (abrasion* or deposit* or disease* or disorder* or erosion* or injur* or oedema* or opacity or opacification or perforation* or ulceration*)) or keratitis or keratitides or Keratoconus).tw,kf. |
| 8 | ((eye adj2 infection*) or Endophthalmitis or Endophthalmitides or (conjunctival adj2 (disease* or injur* or ulceration*)) or Conjunctivitis or Conjunctivitides or "pink eye" or Hordeola or Hordeolum or Keratoconjunctivitis).tw,kf. |
| 9 | (((Eye or eyelid or conjunctival or intraocular or orbital or retinal or uveal or choroid or choroidal or iris or ocular or periocular) adj3 (neoplasm* or cancer* or tumo?r* or lymphoma* or glioma* or carcinoma* or metastases)) or "retinal neuroblastoma*" or "retinal glioblastoma" or retinoblastoma* or "Ocular Paraneoplastic Syndrome*").tw,kf. |
| 10 | ((eye or Ophthalmic or Ophthalmological) adj2 (discharge* or disease* or disorder* or edema or oedema or malformation* or manifestation* or pain or swelling)).tw,kf. |
| 11 | ((eyelid adj2 (disease* or disorder* or pain or retraction*)) or Blepharitis or Blepharitides or Blepharospasm* or Blepharophimosis or Blepharoptosis or Chalazion or Chalazia or "Meibomian Cyst*" or Ectropion or Entropion or Trichias#s or "Periorbital Fat Herniation*").tw,kf. |
| 12 | ("fixed pupil*" or Anisocoria or Miosis or "Horner* Syndrome*" or Mydriasis or "Tonic Pupil*" or Mydriases or "pupil disorder*").tw,kf. |
| 13 | ((ocular adj3 (hypertension* or hypotension* or discomfort* or degeneration or disease* or herpes or infection* or inflammation* or injur* or disorder*)) or glaucoma*).tw,kf. |
| 14 | (Uveitis or iritis or Uveitides or Iritides or ((iris or uveal) adj2 (disease* or disorder*)) or "Posterior Synechiae").tw,kf. |
| 15 | ((lens adj4 (disease* or disorder* or opacit* or subluxation* or dislocation*)) or Aphakia* or Cataract*).tw,kf. |
| 16 | ((macular adj2 (degeneration or hole* or oedema* or edema*)) or maculopath*).tw,kf. |
| 17 | ((retinal adj2 (edema* or oedema* or defect* or deposit* or detachment* or disease* or disorder* tear* or thrombosis or occlusion*)) or retinitis or retinitides or retinoblastoma* or retinopath*).tw,kf. |
| 18 | "Accommodation, Ocular"/ |
| 19 | ((abnormal adj3 (accommodation or accommodative)) or (accommodative adj3 (disorder* or dysfunction*))).tw,kf. |
| 20 | "Ocular Convergence"/ |
| 21 | ((binocular or ocular or eye) adj2 convergence*).tw,kf. |
| 22 | or/1-21 |
| 23 | exp "Ophthalmic SOlutions"/ |
| 24 | ("eye drop" or "eye drops" or "Ophthalmic Solution*" or eyedrop*).tw,kf. |
| 25 | exp "Eye Protective Devices"/ |
| 26 | ((eye adj2 protective adj2 (wear or device)) or "protective eyewear" or "protective eye wear" or (safety adj2 (goggle* or lense* or glasses or lens))).tw,kf. |
| 27 | "organ transplantation"/ and exp "eye"/ |
| 28 | (eye adj2 transplant*).tw,kf. |
| 29 | exp "Injections, Intraocular"/ |
| 30 | ((intraocular or ocular or periocular or eye) adj2 injection*).tw,kf. |
| 31 | exp "Ophthalmologic Surgical Procedures"/ |
| 32 | (LASIK or LASEK or (ophthalmologic adj4 (surger* or procedure*)) or Vitrectom* or "capsular tension ring*" or "intraocular lens implant*" or (cornea adj2 (implant* or transplant*)) or "eyelid inlay*" or "eyelid spacer*" or ((intravitreal or retinal or sclerectomy) adj2 (implant* or transplant*)) or "lacrimal stent*" or "palpebral spring*" or "punctal plug*" or "ab interno gel implant*" or "ab interno gel stent*" or "anterior chamber drainage tube*" or (aqueous adj3 (device* or implant* or shunt*)) or glaukos or istent).tw,kf. |
| 33 | exp "Administration, Ophthalmic"/ |
| 34 | ((ophthalmic or ocular or "intra ocular" or intraocular) adj3 (administration* or delivery or dosage or medication* or treatment*)).tw,kf. |
| 35 | exp "Diagnostic Techniques, Ophthalmological"/ |
| 36 | ((ophthalmologic* adj2 (diagnos#s or diagnostic*)) or "vision test*" or "vision screen*" or retinoscop* or ophthalmoscop*).tw,kf. |
| 37 | exp "Lenses"/ |
| 38 | ("optical device*" or ((eye or contact or corrective or iris or ocular or intraocular or prosthetic or scleral or corneal) adj3 (lense* or lens)) or eyeglass* or "eye glass*" or spectacle* or glasses or "artificial iris" or "lens implant*" or "lens transplant*" or "artificial lens").tw,kf. |
| 39 | exp "Orbital Implants"/ or exp "Orthoptics"/ |
| 40 | ((orbital adj2 implant*) or Orthoptic*).tw,kf. |
| 41 | exp "Visual Prosthesis"/ |
| 42 | (((retinal or visual) adj2 prosthes#s) or "bionic eye*" or "artificial eye*" or keratoprosthes#s or "ocular cosmetic shell*").tw,kf. |
| 43 | exp "Glaucoma Drainage Implants"/ |
| 44 | Pseudophakia/ |
| 45 | (Pseudophakia or pseudophakos).tw,kf. |
| 46 | or/23-45 |
| 47 | 22 or 46 |
| 48 | exp "Social Determinants of Health"/ or *Social Change/ or *Social Welfare/ or exp Healthcare Disparities/ or exp Health Status Disparities/ or *Residence Characteristics/ or *Sociological Factors/ or exp Health Equity/ |
| 49 | ("social determinant of health" or "social determinants of health" or "sdoh" or "social determining factor*" or "social factor* in health" or "social health determinant*" or "social risk factor" or "social determinant*" or "health determinant*" or "basic need*" or "social need*" or "social services" or "social structural determinant*" or "social injustice").tw,kf. |
| 50 | ((healthcare or health) adj2 (equit* or inequit* or disparit* or equalit* or inequalit*)).tw,kf. |
| 51 | 48 or 49 or 50 |
| 52 | exp Social Class/ or *Social Problems/ or exp Socioeconomic Factors/ or *Models, Economic/ or *Economic Status/ or *Unemployment/ or *Employment/ or *Income/ or *Homeless Persons/ or *Housing/ or exp Public Housing/ or exp Public Assistance/ or exp Poverty/ or exp Food Assistance/ or *Food Services/ or *Food Supply/ or *Hunger/ |
| 53 | ("economic stability" or unemployment or employment or "job security" or "economic opportunit*" or "low income" or debt or bankruptcy or "social class*" or "socioeconomic status" or "socioeconomic factor*" or "socioeconomic disadvantage*" or "socioeconomic inequalit*" or "economic disparit*" or "economic disadvantage*" or "financial instability" or "resource poor" or "social status" or "social condition*" or poverty or "lower class" or "middle class" or "economic status" or "economic factor*" or housing or lodging* or domicile* or "living arrangement*" or residence* or dwelling* or homeless or homelessness or "living accommodation*" or residential or "rental assistance" or "rent assistance" or "public assistance" or "food stress" or "food insecur*" or "food hardship" or "food insufficienc*" or "food assistance" or "food suppl*" or "food security*" or "food aid" or "supplemental nutrition assistance" or SNAP or "food stamp*" or "WIC program*" or "women infants and children program" or eviction* or bankruptcy or foreclosure or mortgage or rural*).tw,kf. |
| 54 | 52 or 53 |
| 55 | exp Social Environment/ or exp Social Support/ or *Psychosocial Support Systems/ or *Psychosocial Deprivation/ or *Social Isolation/ or exp Racism/ or exp Prejudice/ or *Social integration/ or exp Violence/ or exp Race Relations/ or *Refugees/ or *working poor/ or exp Social Stigma/ or *social control, informal/ or exp Social networking/ |
| 56 | (social adj2 (context or environment or ecolog* or integrat* or isolat* or trust or vulnerability or cohes* or capital or organi?ation)).tw,kf. |
| 57 | (racial adj2 (discriminat* or bias or prejudice or cohes*)).tw,kf. |
| 58 | ((community or civic) adj2 (engagement or cooperation or context or cohes* or violence or "sense of")).tw,kf. |
| 59 | (violence adj2 (exposure or experience* or neighbo?hood or witness* or victim)).tw,kf. |
| 60 | (racism or segregation or "ethnic group*").tw,kf. |
| 61 | 55 or 56 or 57 or 58 or 59 or 60 |
| 62 | exp Environmental Exposure/ or *Environment Design/ or exp Built Environment/ or exp Urban renewal/ or exp Parks, Recreational/ or exp Transportation/ |
| 63 | (transportation or commute or "public transit" or highway* or subway or "mixed use" or pedestrian or walkab*).tw,kf. |
| 64 | ("green space*" or park$1 or playground* or sidewalk* or "community garden$1" or "food desert" or "grocery store density" or "fast food" or "restaurant density" or "healthy food availability" or HFAI or "nutrition environment measures" or NEMS).tw,kf. |
| 65 | ((owner adj2 (vehicle or auto* or car)) or "alcohol outlet*" or neighbo?rhood or "housing quality" or "urban environment" or "inner cit*").tw,kf. |
| 66 | 62 or 63 or 64 or 65 |
| 67 | exp "Educational Status"/ or exp Literacy/ or exp Education/ |
| 68 | (litera$2 adj2 (health or cancer or English)).tw,kf. |
| 69 | (degree adj2 (university or college or advanced or "high school")).tw,kf. |
| 70 | (GED or "high school graduate" or "high school dropout" or "high school completion").tw,kf. |
| 71 | (educational* adj2 (level? or status or attainment or achievement or access)).tw,kf. |
| 72 | 67 or 68 or 69 or 70 or 71 |
| 73 | exp Cultural Competency/ or exp Culturally Competent Care/ or exp Patient Navigation/ or *acculturation/ or exp Cross-Cultural Comparison/ or exp Cultural Diversity/ or exp Health Services Accessibility/ or *medicare/ or *Medicaid/ or exp Medically Uninsured/ or exp Insurance Coverage/ or exp Insurance, Health/ |
| 74 | ("patient navigat*" or "transcultural care" or "culturally appropriate care" or "culturally competent care" or "cultural care" or "cultural competenc*").tw,kf. |
| 75 | ((healthcare or care or provider or "health services" or insurance) adj2 (access* or availab*)).tw,kf. |
| 76 | 73 or 74 or 75 |
| 77 | 51 or 54 or 61 or 66 or 72 or 76 |
| 78 | 47 and 77 |
| 79 | 78 not (exp animals/ not exp humans/) |
| 80 | 79 and English.lg. |
| 81 | ("United States" or America or American).af. |
| 82 | 80 and 81 |
| 83 | limit 82 to yr=2000-current |

| Embase (via Elsevier, Embase.com, 1947 to November 23, 2024) | |
| --- | --- |
| **#** | **Searches** |
| 79 | #78 NOT 'conference abstract':it |
| 78 | #77 AND [01-01-2020]/sd |
| 77 | #75 AND #76 |
| 76 | 'united states' OR america OR american |
| 75 | #74 AND english:la |
| 74 | #73 NOT ([animals]/lim NOT [humans]/lim) |
| 73 | #42 AND #72 |
| 72 | #46 OR #49 OR #56 OR #61 OR #67 OR #71 |
| 71 | #68 OR #69 OR #70 |
| 70 | ((healthcare OR care OR provider OR 'health services' OR insurance) NEAR/2 (access* OR availab*)):ti,ab,kw |
| 69 | 'patient navigat*':ti,ab,kw OR 'transcultural care':ti,ab,kw OR 'culturally appropriate care':ti,ab,kw OR 'culturally competent care':ti,ab,kw OR 'cultural care':ti,ab,kw OR 'cultural competenc*':ti,ab,kw |
| 68 | 'cultural competence'/exp OR 'transcultural care'/exp OR 'cultural factor'/exp/mj OR 'cultural diversity'/exp OR 'health care access'/exp OR 'medicare'/mj OR 'medicaid'/mj OR 'medically uninsured'/exp OR 'insurance'/de OR 'health insurance'/exp |
| 67 | #62 OR #63 OR #64 OR #65 OR #66 |
| 66 | (educational* NEAR/2 (level OR status OR attainment OR achievement OR access)):ti,ab |
| 65 | ged:ti,ab,kw OR 'high school graduate':ti,ab,kw OR 'high school dropout':ti,ab,kw OR 'high school completion':ti,ab,kw |
| 64 | (degree NEAR/2 (university OR college OR advanced OR 'high school')):ti,ab |
| 63 | ((literate OR literacy) NEAR/2 (health OR english)):ti,ab,kw |
| 62 | 'educational status'/mj OR 'literacy'/exp/mj OR 'education'/exp/mj |
| 61 | #57 OR #58 OR #59 OR #60 |
| 60 | ((owner NEAR/2 (vehicle OR auto* OR car)):ti,ab,kw) OR 'alcohol outlet*':ti,ab,kw OR neighbo$rhood:ti,ab,kw OR 'housing quality':ti,ab,kw OR 'urban environment':ti,ab,kw OR 'inner cit*':ti,ab,kw |
| 59 | 'green space*':ti,ab,kw OR park?1:ti,ab,kw OR playground*:ti,ab,kw OR sidewalk*:ti,ab,kw OR 'community garden?1':ti,ab,kw OR 'food desert':ti,ab,kw OR 'grocery store density':ti,ab,kw OR 'fast food':ti,ab,kw OR 'restaurant density':ti,ab,kw OR 'healthy food availability':ti,ab,kw OR hfai:ti,ab,kw OR 'nutrition environment measures':ti,ab,kw OR nems:ti,ab,kw |
| 58 | transportation:ti,ab,kw OR commute:ti,ab,kw OR 'public transit':ti,ab,kw OR highway*:ti,ab,kw OR subway:ti,ab,kw OR 'mixed use':ti,ab,kw OR pedestrian:ti,ab,kw OR walkab*:ti,ab,kw |
| 57 | 'environmental exposure'/exp OR 'environmental planning'/exp/mj OR 'built environment'/exp OR 'city planning'/exp OR 'recreational park'/exp OR 'traffic and transport'/exp/mj |
| 56 | #50 OR #51 OR #52 OR #53 OR #54 OR #55 |
| 55 | racism:ti,ab,kw OR segregation:ti,ab OR 'ethnic group*':ti,ab |
| 54 | (violence NEAR/2 (exposure OR experience* OR neighbo$hood OR witness* OR victim)):ti,ab,kw |
| 53 | ((community OR civic) NEAR/2 (engagement OR cooperation OR context OR cohes* OR violence OR 'sense of')):ti,ab,kw |
| 52 | (racial NEAR/2 (discriminat* OR bias OR prejudice OR cohes*)):ti,ab,kw |
| 51 | (social NEXT/2 (context OR environment OR ecolog* OR integrat* OR isolat* OR trust OR vulnerability OR cohes* OR capital OR organi$ation)):ti,ab,kw |
| 50 | 'social environment'/exp/mj OR 'social support'/exp/mj OR 'psychosocial care'/mj OR 'social isolation'/exp/mj OR 'racism'/exp OR 'prejudice'/exp OR 'social integration'/exp/mj OR 'violence'/exp/mj OR 'race relation'/exp OR 'refugee'/exp/mj OR 'working poor'/mj OR 'social stigma'/exp OR 'social control'/exp/mj OR 'social network'/exp |
| 49 | #47 OR #48 |
| 48 | 'economic stability':ti,ab OR unemployment:ti,ab OR employment:ti,ab OR 'job security':ti,ab OR 'economic opportunit*':ti,ab OR 'low income':ti,ab OR debt:ti,ab OR 'social class*':ti,ab OR 'socioeconomic status':ti,ab OR 'socioeconomic factor*':ti,ab OR 'socioeconomic disadvantage*':ti,ab OR 'socioeconomic inequalit*':ti,ab OR 'economic disparit*':ti,ab OR 'economic disadvantage*':ti,ab OR 'financial instability':ti,ab OR 'resource poor':ti,ab OR 'social status':ti,ab OR 'social condition*':ti,ab OR poverty:ti,ab OR 'lower class':ti,ab OR 'middle class':ti,ab OR 'economic status':ti,ab OR 'economic factor*':ti,ab OR housing:ti,ab OR lodging*:ti,ab OR domicile*:ti,ab OR 'living arrangement*':ti,ab OR residence*:ti,ab OR dwelling*:ti,ab OR homeless:ti,ab OR homelessness:ti,ab OR 'ill-housed':ti,ab OR 'living accommodation*':ti,ab OR residential:ti,ab OR 'rental assistance':ti,ab OR 'rent assistance':ti,ab OR 'public assistance':ti,ab OR 'food stress':ti,ab OR 'food insecurity':ti,ab OR 'food insecur*':ti,ab OR 'food hardship':ti,ab OR 'food insufficienc*':ti,ab OR 'food assistance':ti,ab OR 'food suppl*':ti,ab OR 'food security*':ti,ab OR 'food aid':ti,ab OR 'supplemental nutrition assistance':ti,ab OR snap:ti,ab OR 'food stamp*':ti,ab OR 'wic program*':ti,ab OR 'women infants and children program':ti,ab OR eviction*:ti,ab OR bankruptcy:ti,ab OR foreclosure:ti,ab OR mortgage:ti,ab OR rural*:ti,ab |
| 47 | 'social class'/exp OR 'social problem'/mj OR 'socioeconomics'/mj OR 'economic model'/mj OR 'economic status'/mj OR 'household economic status'/mj OR 'unemployment'/mj OR 'employment'/mj OR 'income'/mj OR 'homeless persons'/exp/mj OR 'homelessness'/mj OR 'housing'/mj OR 'housing instability'/exp OR 'social care'/exp OR 'poverty'/exp OR 'food assistance'/exp OR 'hunger'/mj OR 'displacement(people)'/mj |
| 46 | #43 OR #44 OR #45 |
| 45 | ((healthcare OR health) NEAR/2 (equit* OR inequit* OR disparit* OR equalit* OR inequalit*)):ti,ab,kw |
| 44 | 'social determinant of health':ti,ab,kw OR 'social determinants of health':ti,ab,kw OR sdoh:ti,ab,kw OR 'social determining factor*':ti,ab,kw OR 'social factor* in health':ti,ab,kw OR 'social health determinant*':ti,ab,kw OR 'social risk factor':ti,ab,kw OR 'social determinant*':ti,ab,kw OR 'health determinant*':ti,ab,kw OR 'basic need*':ti,ab,kw OR 'social need*':ti,ab,kw OR 'social services':ti,ab,kw OR 'social structural determinant*':ti,ab,kw OR 'social injustice':ti,ab,kw |
| 43 | 'social determinants of health'/exp OR 'social change'/mj OR 'social welfare'/mj OR 'health care disparity'/exp OR 'health disparity'/exp OR 'residence characteristics'/mj OR 'social aspects and related phenomena'/mj OR 'health equity'/exp |
| 42 | #22 OR #41 |
| 41 | #23 OR #24 OR #25 OR #26 OR #27 OR #28 OR #29 OR #30 OR #31 OR #32 OR #33 OR #34 OR #35 OR #36 OR #37 OR #38 OR #39 OR #40 |
| 40 | (((retinal OR visual) NEAR/2 prosthes?s):ti,ab,kw) OR 'bionic eye*':ti,ab,kw OR 'artificial eye*':ti,ab,kw OR keratoprosthes?s:ti,ab,kw OR 'ocular cosmetic shell*':ti,ab,kw |
| 39 | ((orbital NEAR/2 implant*):ti,ab,kw) OR orthoptic*:ti,ab,kw |
| 38 | 'ophthalmological prosthesis and implant'/exp OR 'orthoptics'/exp |
| 37 | 'optical device*':ti,ab,kw OR (((eye OR contact OR corrective OR iris OR ocular OR intraocular OR prosthetic OR scleral OR corneal) NEAR/3 (lense* OR lens)):ti,ab,kw) OR eyeglass*:ti,ab,kw OR 'eye glass*':ti,ab,kw OR spectacle*:ti,ab,kw OR glasses:ti,ab,kw OR 'artificial iris':ti,ab,kw OR 'lens implant*':ti,ab,kw OR 'lens transplant*':ti,ab,kw OR 'artificial lens':ti,ab,kw |
| 36 | 'visual aid'/exp |
| 35 | ((ophthalmologic* NEAR/2 (diagnos?s OR diagnostic*)):ti,ab,kw) OR 'vision test*':ti,ab,kw OR 'vision screen*':ti,ab,kw OR retinoscop*:ti,ab,kw OR ophthalmoscop*:ti,ab,kw |
| 34 | 'visual system examination'/exp |
| 33 | lasik:ti,ab,kw OR lasek:ti,ab,kw OR ((ophthalmologic NEAR/4 (surger* OR procedure*)):ti,ab,kw) OR vitrectom*:ti,ab,kw OR 'capsular tension ring*':ti,ab,kw OR 'intraocular lens implant*':ti,ab,kw OR ((cornea NEAR/2 (implant* OR transplant*)):ti,ab,kw) OR 'eyelid inlay*':ti,ab,kw OR 'eyelid spacer*':ti,ab,kw OR (((intravitreal OR retinal OR sclerectomy) NEAR/2 (implant* OR transplant*)):ti,ab,kw) OR 'lacrimal stent*':ti,ab,kw OR 'palpebral spring*':ti,ab,kw OR 'punctal plug*':ti,ab,kw OR 'ab interno gel implant*':ti,ab,kw OR 'ab interno gel stent*':ti,ab,kw OR 'anterior chamber drainage tube*':ti,ab,kw OR ((aqueous NEAR/3 (device* OR implant* OR shunt*)):ti,ab,kw) OR glaukos:ti,ab,kw OR istent:ti,ab,kw |
| 32 | 'eye surgery'/exp |
| 31 | ((ophthalmic OR ocular OR 'intra ocular' OR intraocular) NEAR/3 (administration* OR delivery OR dosage OR medication* OR treatment*)):ti,ab,kw |
| 30 | ((intraocular OR ocular OR periocular OR eye) NEAR/2 injection*):ti,ab,kw |
| 29 | 'intraocular drug administration'/exp |
| 28 | (eye NEAR/2 transplant*):ti,ab,kw |
| 27 | 'eye transplantation'/exp |
| 26 | ((eye NEAR/2 protective NEAR/2 (wear OR device)):ti,ab,kw) OR 'protective eyewear':ti,ab,kw OR 'protective eye wear':ti,ab,kw OR ((safety NEAR/2 (goggle* OR lense* OR glasses OR lens)):ti,ab,kw) |
| 25 | 'eye protective device'/exp |
| 24 | 'eye drop':ti,ab,kw OR 'eye drops':ti,ab,kw OR 'ophthalmic solution*':ti,ab,kw OR eyedrop*:ti,ab,kw |
| 23 | 'eye drops'/exp |
| 22 | #1 OR #2 OR #3 OR #4 OR #5 OR #6 OR #7 OR #8 OR #9 OR #10 OR #11 OR #12 OR #13 OR #14 OR #15 OR #16 OR #17 OR #18 OR #19 OR #20 OR #21 |
| 21 | ((ocular OR binocular OR eye) NEAR/2 convergence*):ti,ab,kw |
| 20 | 'binocular convergence'/exp |
| 19 | ((abnormal NEAR/3 (accommodation OR accommodative)):ti,ab,kw) OR ((accommodative NEAR/3 (disorder* OR dysfunction*)):ti,ab,kw) |
| 18 | 'accommodation'/exp |
| 17 | ((retinal NEAR/2 (edema* OR oedema* OR defect* OR deposit* OR detachment* OR disease* OR 'disorder* tear*' OR thrombosis OR occlusion*)):ti,ab,kw) OR retinitis:ti,ab,kw OR retinitides:ti,ab,kw OR retinoblastoma*:ti,ab,kw OR retinopath*:ti,ab,kw |
| 16 | ((macular NEAR/2 (degeneration OR hole* OR oedema* OR edema*)):ti,ab,kw) OR maculopath*:ti,ab,kw |
| 15 | ((lens NEAR/4 (disease* OR disorder* OR opacit* OR subluxation* OR dislocation*)):ti,ab,kw) OR aphakia*:ti,ab,kw OR cataract*:ti,ab,kw |
| 14 | uveitis:ti,ab,kw OR iritis:ti,ab,kw OR uveitides:ti,ab,kw OR iritides:ti,ab,kw OR (((iris OR uveal) NEAR/2 (disease* OR disorder*)):ti,ab,kw) OR 'posterior synechiae':ti,ab,kw |
| 13 | ((ocular NEAR/3 (hypertension* OR hypotension* OR discomfort* OR degeneration OR disease* OR herpes OR infection* OR inflammation* OR injur* OR disorder*)):ti,ab,kw) OR glaucoma*:ti,ab,kw |
| 12 | 'fixed pupil*':ti,ab,kw OR anisocoria:ti,ab,kw OR miosis:ti,ab,kw OR 'horner* syndrome*':ti,ab,kw OR mydriasis:ti,ab,kw OR 'tonic pupil*':ti,ab,kw OR mydriases:ti,ab,kw OR 'pupil disorder*':ti,ab,kw |
| 11 | ((eyelid NEAR/2 (disease* OR disorder* OR pain OR retraction*)):ti,ab,kw) OR blepharitis:ti,ab,kw OR blepharitides:ti,ab,kw OR blepharospasm*:ti,ab,kw OR blepharophimosis:ti,ab,kw OR blepharoptosis:ti,ab,kw OR chalazion:ti,ab,kw OR chalazia:ti,ab,kw OR 'meibomian cyst*':ti,ab,kw OR ectropion:ti,ab,kw OR entropion:ti,ab,kw OR trichias?s:ti,ab,kw OR 'periorbital fat herniation*':ti,ab,kw |
| 10 | ((eye OR ophthalmic OR ophthalmological) NEAR/2 (discharge* OR disease* OR disorder* OR edema OR oedema OR malformation* OR manifestation* OR pain OR swelling)):ti,ab,kw |
| 9 | (((eye OR eyelid OR conjunctival OR intraocular OR orbital OR retinal OR uveal OR choroid OR choroidal OR iris OR ocular OR periocular) NEAR/3 (neoplasm* OR cancer* OR tumo$r* OR lymphoma* OR glioma* OR carcinoma* OR metastases)):ti,ab,kw) OR 'retinal neuroblastoma*':ti,ab,kw OR 'retinal glioblastoma':ti,ab,kw OR retinoblastoma*:ti,ab,kw OR 'ocular paraneoplastic syndrome*':ti,ab,kw |
| 8 | ((eye NEAR/2 infection*):ti,ab,kw) OR endophthalmitis:ti,ab,kw OR endophthalmitides:ti,ab,kw OR ((conjunctival NEAR/2 (disease* OR injur* OR ulceration*)):ti,ab,kw) OR conjunctivitis:ti,ab,kw OR conjunctivitides:ti,ab,kw OR 'pink eye':ti,ab,kw OR hordeola:ti,ab,kw OR hordeolum:ti,ab,kw OR keratoconjunctivitis:ti,ab,kw |
| 7 | ((eye NEAR/2 (injur* OR burn* OR 'foreign bodies' OR 'foreign body' OR discharge*)):ti,ab,kw) OR 'traumatic hyphema*':ti,ab,kw OR ((corneal NEAR/2 (abrasion* OR deposit* OR disease* OR disorder* OR erosion* OR injur* OR oedema* OR opacity OR opacification OR perforation* OR ulceration*)):ti,ab,kw) OR keratitis:ti,ab,kw OR keratitides:ti,ab,kw OR keratoconus:ti,ab,kw |
| 6 | eyestrain:ti,ab OR 'eye fatigue':ti,ab OR 'eye strain':ti,ab OR 'visual fatigue':ti,ab OR 'eye redness':ti,ab |
| 5 | ((visual NEAR/3 (loss OR agnosia* OR defect* OR difficult* OR disorder* OR disturbance* OR constriction* OR impair*)):ti,ab,kw) OR (((loss OR abnormal OR blurred OR decreased OR defect* OR deficienc* OR disorder* OR dim OR difficulty OR diminished OR disturbed OR disturbance OR hazy OR impair* OR interference OR reduced OR weak OR partial OR subnormal OR tunnel OR problem) NEAR/2 vision):ti,ab,kw) OR hypermetropia:ti,ab,kw OR farsighted*:ti,ab,kw OR 'far sighted':ti,ab,kw OR myopia:ti,ab,kw OR nearsighted*:ti,ab,kw OR 'near sighted':ti,ab,kw OR blindness:ti,ab,kw OR 'legal* blind*':ti,ab,kw OR 'delayed visual maturation':ti,ab,kw OR 'difficulty seeing':ti,ab,kw OR 'low vision':ti,ab,kw OR 'impaired visual acuity':ti,ab,kw OR 'partially sighted':ti,ab,kw OR (((impair* OR partial) NEAR/2 sight):ti,ab,kw) OR ((sunken NEAR/2 (eye OR eyes OR eyelid)):ti,ab,kw) OR symblepharon:ti,ab,kw |
| 4 | 'dry eye':ti,ab,kw OR 'dry eyes':ti,ab,kw OR ((lacrimal NEAR/3 (disorder* OR disease* OR obstruction*)):ti,ab,kw) OR (((decreased OR abnormal) NEAR/3 lacrimation):ti,ab,kw) OR 'meibomian gland dysfunction*':ti,ab,kw OR 'mg dysfunction*':ti,ab,kw OR meibomianitis:ti,ab,kw OR meibomianitides:ti,ab,kw OR xerophthalmia*:ti,ab,kw |
| 3 | anophthalmos:ti,ab,kw OR anophthalmia*:ti,ab,kw OR ((eye NEAR/2 abnormalit*):ti,ab,kw) OR vitreoretinopath*:ti,ab,kw OR hydrophthalmos:ti,ab,kw OR microphthalmos:ti,ab,kw OR 'retinal dysplasia*':ti,ab,kw |
| 2 | (((refractive OR refraction) NEAR/2 (error* OR disorder*)):ti,ab,kw) OR 'wavefront aberration*':ti,ab,kw OR amblyopia*:ti,ab,kw OR 'lazy eye':ti,ab,kw OR amtropia*:ti,ab,kw OR aniseikonia:ti,ab,kw OR anisometropia*:ti,ab,kw OR astigmatism*:ti,ab,kw OR ((corneal NEAR/3 abberation*):ti,ab,kw) OR hyperopia:ti,ab,kw OR presbyopia:ti,ab,kw OR (((eye OR vitreous OR 'anterior chamber' OR choroid OR retinal OR conjunctival OR ocular) NEAR/2 h$emorrhage):ti,ab,kw) OR hyphema*:ti,ab,kw OR ((aqueous NEAR/2 'outflow obstruction*'):ti,ab,kw) OR asthenopia:ti,ab,kw OR 'balint* syndrome':ti,ab,kw OR hemianopia*:ti,ab,kw OR hemianopsia*:ti,ab,kw OR pseudoaphakia*:ti,ab,kw OR 'capsule opacification*':ti,ab,kw OR ((chorioretinal NEAR/2 (disorder* OR disease*)):ti,ab,kw) OR chorioretinitis:ti,ab,kw OR chorioretinitides:ti,ab,kw OR 'choroid disease*':ti,ab,kw OR choroidal:ti,ab,kw OR choroiditis:ti,ab,kw OR choroiditides:ti,ab,kw OR chromatopsia:ti,ab,kw OR epiphora*:ti,ab,kw OR diplopia*:ti,ab,kw OR polyopsia*:ti,ab,kw OR ophthalmia*:ti,ab,kw OR episcleritis:ti,ab,kw OR scleritis:ti,ab,kw OR ((scleral NEAR/2 disease*):ti,ab,kw) OR (((equatorial OR scleral OR anterior OR posticum) NEAR/2 staphyloma):ti,ab,kw) OR strabismus:ti,ab,kw OR exotropia:ti,ab,kw OR esotropia*:ti,ab,kw OR esodeviation*:ti,ab,kw OR 'orbital disease*':ti,ab,kw OR enophthalmos:ti,ab,kw OR exophthalmos:ti,ab,kw OR proptos$s:ti,ab,kw OR ophthalmopath*:ti,ab,kw OR ophthalmoplegia*:ti,ab,kw OR 'eye muscle paralysis':ti,ab,kw OR ((fuchs NEAR/3 (dystrophy OR atrophy)):ti,ab,kw) OR nystagmus:ti,ab,kw OR oculopath*:ti,ab,kw OR ((optic NEAR/3 (atrophy OR disease* OR disorder* OR glioma* OR injur* OR neuritis OR papillitis)):ti,ab,kw) OR papilledema*:ti,ab,kw OR 'optic disk edema*':ti,ab,kw OR 'optic oedema*':ti,ab,kw OR 'choked disk*':ti,ab,kw OR photalgia:ti,ab,kw OR photophobia*:ti,ab,kw OR photopsia*:ti,ab,kw OR 'retrobulbar neuritis':ti,ab,kw OR scotoma*:ti,ab,kw OR ((vitreous NEAR/2 (detachment* OR strand* OR vein* OR prolapse* OR syneresis OR pseudophakia OR pseudophakos)):ti,ab,kw) |
| 1 | 'eye disease'/exp OR 'eye injury'/exp OR 'eye malformation'/exp OR 'hemianopia'/exp |

| Web of Science Core Collection (via Clarivate Analytics, including Science Citation Index Expanded and Social Sciences Citation Index, 1974 to November 23, 2024; and Emerging Sources Citation Index from 2005 to November 23, 2024) | |
| --- | --- |
| **#** | **Searches** |
| 1 | TS=(((refractive OR refraction) NEAR/2 (error* OR disorder*)) OR "wavefront aberration*" OR Amblyopia* OR "lazy eye" OR amtropia* OR Aniseikonia OR Anisometropia* OR astigmatism* OR (corneal NEAR/3 abberation*) OR Hyperopia OR Presbyopia OR ((eye OR vitreous OR "anterior chamber" OR choroid OR retinal OR conjunctival OR ocular) NEAR/2 h$emorrhage) OR Hyphema* OR (aqueous NEAR/2 "outflow obstruction*") OR Asthenopia OR "Balint* Syndrome" OR Hemianopia* OR Hemianopsia* OR Pseudoaphakia* OR "capsule opacification*" OR (Chorioretinal NEAR/2 (Disorder* OR disease*)) OR Chorioretinitis OR Chorioretinitides OR "Choroid Disease*" OR Choroidal OR Choroiditis OR Choroiditides OR Chromatopsia OR Epiphora* OR Diplopia* OR Polyopsia* OR Ophthalmia* OR Episcleritis OR Scleritis OR (scleral NEAR/2 disease*) OR ((equatorial OR scleral OR anterior OR posticum) NEAR/2 staphyloma) OR strabismus OR exotropia OR Esotropia* OR Esodeviation* OR "orbital disease*" OR enophthalmos OR Exophthalmos OR Proptos$s OR ophthalmopath* OR Ophthalmoplegia* OR "eye muscle paralysis" OR (Fuchs NEAR/3 (dystrophy OR atrophy)) OR Nystagmus OR Oculopath* OR (optic NEAR/3 (atrophy OR disease* OR disorder* OR glioma* OR injur* OR neuritis OR papillitis)) OR Papilledema* OR "optic disk edema*" OR "optic oedema*" OR "choked disk*" OR Photalgia OR Photophobia* OR Photopsia* OR "Retrobulbar Neuritis" OR scotoma* OR (vitreous NEAR/2 (detachment* OR strand* OR vein* OR prolapse* OR syneresis))) |
| 2 | TS=(Anophthalmos OR Anophthalmia* OR (eye NEAR/2 abnormalit*) OR Vitreoretinopath* OR Hydrophthalmos OR Microphthalmos OR "retinal dysplasia*") |
| 3 | TS=("dry eye" OR "dry eyes" OR (lacrimal NEAR/3 (disorder* OR disease* OR obstruction*)) OR ((decreased OR abnormal) NEAR/3 lacrimation) OR "Meibomian Gland Dysfunction*" OR "MG Dysfunction*" OR Meibomianitis OR Meibomianitides OR Xerophthalmia*) |
| 4 | TS=((visual NEAR/3 (loss OR agnosia* OR defect* OR difficult* OR disorder* OR disturbance* OR constriction* OR impair*)) OR ((loss OR abnormal OR blurred OR decreased OR defect* OR deficienc* OR disorder* OR dim OR difficulty OR diminished OR disturbed OR disturbance OR hazy OR impair* OR interference OR reduced OR weak OR partial OR subnormal OR tunnel OR problem) NEAR/2 vision) OR Hypermetropia OR Farsighted* OR "far sighted" OR Myopia OR nearsighted* OR "near sighted" OR Blindness OR "legal* blind*" OR "Delayed Visual Maturation" OR "difficulty seeing" OR "low vision" OR "impaired visual acuity" OR "partially sighted" OR ((impair* OR partial) NEAR/2 sight) OR (sunken NEAR/2 (eye OR eyes OR eyelid)) OR Symblepharon) |
| 5 | (TI=(eyestrain OR "eye fatigue" OR "eye strain" OR "visual fatigue" OR "eye redness") OR AB=(eyestrain OR "eye fatigue" OR "eye strain" OR "visual fatigue" OR "eye redness")) |
| 6 | TS=((eye NEAR/2 (injur* OR Burn* OR "foreign bodies" OR "foreign body" OR discharge*)) OR "traumatic hyphema*" OR (corneal NEAR/2 (abrasion* OR deposit* OR disease* OR disorder* OR erosion* OR injur* OR oedema* OR opacity OR opacification OR perforation* OR ulceration*)) OR keratitis OR keratitides OR Keratoconus) |
| 7 | TS=((eye NEAR/2 infection*) OR Endophthalmitis OR Endophthalmitides OR (conjunctival NEAR/2 (disease* OR injur* OR ulceration*)) OR Conjunctivitis OR Conjunctivitides OR "pink eye" OR Hordeola OR Hordeolum OR Keratoconjunctivitis) |
| 8 | TS=(((Eye OR eyelid OR conjunctival OR intraocular OR orbital OR retinal OR uveal OR choroid OR choroidal OR iris OR ocular OR periocular) NEAR/3 (neoplasm* OR cancer* OR tumo$r* OR lymphoma* OR glioma* OR carcinoma* OR metastases)) OR "retinal neuroblastoma*" OR "retinal glioblastoma" OR retinoblastoma* OR "Ocular Paraneoplastic Syndrome*") |
| 9 | TS=((eye OR Ophthalmic OR Ophthalmological) NEAR/2 (discharge* OR disease* OR disorder* OR edema OR oedema OR malformation* OR manifestation* OR pain OR swelling)) |
| 10 | TS=((eyelid NEAR/2 (disease* OR disorder* OR pain OR retraction*)) OR Blepharitis OR Blepharitides OR Blepharospasm* OR Blepharophimosis OR Blepharoptosis OR Chalazion OR Chalazia OR "Meibomian Cyst*" OR Ectropion OR Entropion OR Trichias?s OR "Periorbital Fat Herniation*") |
| 11 | TS=("fixed pupil*" OR Anisocoria OR Miosis OR "Horner* Syndrome*" OR Mydriasis OR "Tonic Pupil*" OR Mydriases OR "pupil disorder*") |
| 12 | TS=((ocular NEAR/3 (hypertension* OR hypotension* OR discomfort* OR degeneration OR disease* OR herpes OR infection* OR inflammation* OR injur* OR disorder*)) OR glaucoma*) |
| 13 | TS=(Uveitis OR iritis OR Uveitides OR Iritides OR ((iris OR uveal) NEAR/2 (disease* OR disorder*)) OR "Posterior Synechiae") |
| 14 | TS=((lens NEAR/4 (disease* OR disorder* OR opacit* OR subluxation* OR dislocation*)) OR Aphakia* OR Cataract*) |
| 15 | TS=((macular NEAR/2 (degeneration OR hole* OR oedema* OR edema*)) OR maculopath*) |
| 16 | TS=((retinal NEAR/2 (edema* OR oedema* OR defect* OR deposit* OR detachment* OR disease* OR "disorder* tear*" OR thrombosis OR occlusion*)) OR retinitis OR retinitides OR retinoblastoma* OR retinopath*) |
| 17 | TS=((abnormal NEAR/3 (accommodation OR accommodative)) OR (accommodative NEAR/3 (disorder* OR dysfunction*))) |
| 18 | TS=((binocular OR ocular OR eye) NEAR/2 convergence*) |
| 19 | #1 OR #2 OR #3 OR #4 OR #5 OR #6 OR #7 OR #8 OR #9 OR #10 OR #11 OR #12 OR #13 OR #14 OR #15 OR #16 OR #17 OR #18 |
| 20 | TI=("eye drop" OR "eye drops" OR "Ophthalmic Solution*" OR eyedrop*) OR AB=("eye drop" OR "eye drops" OR "Ophthalmic Solution*" OR eyedrop*) |
| 21 | TS=((eye NEAR/2 protective NEAR/2 (wear OR device)) OR "protective eyewear" OR "protective eye wear" OR (safety NEAR/2 (goggle* OR lense* OR glasses OR lens))) |
| 22 | TS=(eye NEAR/2 (transplant*)) |
| 23 | TS=((intraocular OR ocular OR periocular OR eye) NEAR/2 injection*) |
| 24 | TS=(LASIK OR LASEK OR (ophthalmologic NEAR/4 (surger* OR procedure*)) OR Vitrectom* OR "capsular tension ring*" OR "intraocular lens implant*" OR (cornea NEAR/2 (implant* OR transplant*)) OR "eyelid inlay*" OR "eyelid spacer*" OR ((intravitreal OR retinal OR sclerectomy) NEAR/2 (implant* OR transplant*)) OR "lacrimal stent*" OR "palpebral spring*" OR "punctal plug*" OR "ab interno gel implant*" OR "ab interno gel stent*" OR "anterior chamber drainage tube*" OR (aqueous NEAR/3 (device* OR implant* OR shunt*)) OR glaukos OR istent) |
| 25 | TS=(((ophthalmic OR ocular OR "intra ocular" OR intraocular) NEAR/3 (administration* OR delivery OR dosage OR medication* OR treatment*))) |
| 26 | TS=((ophthalmologic* NEAR/2 (diagnos?s OR diagnostic*)) OR "vision test*" OR "vision screen*" OR retinoscop* OR ophthalmoscop*) |
| 27 | TS=("optical device*" OR ((eye OR contact OR corrective OR iris OR ocular OR intraocular OR prosthetic OR scleral OR corneal) NEAR/3 (lense* OR lens)) OR eyeglass* OR "eye glass*" OR spectacle* OR glasses OR "artificial iris" OR "lens implant*" OR "lens transplant*" OR "artificial lens") |
| 28 | TS=((orbital NEAR/2 implant*) OR Orthoptic*) |
| 29 | TS=(((retinal OR visual) NEAR/2 prosthes?s) OR "bionic eye*" OR "artificial eye*" OR keratoprosthes?s OR "ocular cosmetic shell*") |
| 30 | TS=(Pseudophakia OR pseudophakos) |
| 31 | #20 OR #21 OR #22 OR #23 OR #24 OR #25 OR #26 OR #27 OR #28 OR #29 OR #30 |
| 32 | #19 OR #31 |
| 33 | TS=("social determinant of health" OR "social determinants of health" OR sdoh OR "social determining factor*" OR "social factor* in health" OR "social health determinant*" OR "social risk factor" OR "social determinant*" OR "health determinant*" OR "basic need*" OR "social need*" OR "social services" OR "social structural determinant*" OR "social injustice") |
| 34 | TS=((healthcare OR health) NEAR/2 (equit* OR inequit* OR disparit* OR equalit* OR inequalit*)) |
| 35 | TS=("economic stability" OR unemployment OR employment OR "job security" OR "economic opportunit*" OR "low income" OR debt OR bankruptcy OR "social class*" OR "socioeconomic status" OR "socioeconomic factor*" OR "socioeconomic disadvantage*" OR "socioeconomic inequalit*" OR "economic disparit*" OR "economic disadvantage*" OR "financial instability" OR "resource poor" OR "social status" OR "social condition*" OR poverty OR "lower class" OR "middle class" OR "economic status" OR "economic factor*" OR housing OR lodging* OR domicile* OR "living arrangement*" OR residence* OR dwelling* OR homeless OR homelessness OR "ill-housed" OR "living accommodation*" OR residential OR "rental assistance" OR "rent assistance" OR "public assistance" OR "food stress" OR "food insecurity" OR "food insecur*" OR "food hardship" OR "food insufficienc*" OR "food assistance" OR "food suppl*" OR "food security*" OR "food aid" OR "supplemental nutrition assistance" OR SNAP OR "food stamp*" OR "WIC program*" OR "women infants and children program" OR eviction* OR bankruptcy OR foreclosure OR mortgage OR rural*) |
| 36 | TS=(social NEAR/2 (context OR environment OR ecolog* OR integrat* OR isolat* OR trust OR vulnerability OR cohes* OR capital OR organi$ation)) |
| 37 | TS=(racial NEAR/2 (discriminat* OR bias OR prejudice OR cohes*)) |
| 38 | TS=((community OR civic) NEAR/2 (engagement OR cooperation OR context OR cohes* OR violence OR "sense of")) |
| 39 | TS=(violence NEAR/2 (exposure OR experience* OR neighbo$hood OR witness* OR victim)) |
| 40 | TS=(racism OR segregation OR "ethnic group*") |
| 41 | TS=(transportation OR commute OR "public transit" OR highway* OR subway OR "mixed use" OR pedestrian OR walkab*) |
| 42 | TS=("green space*" OR park?1 OR playground* OR sidewalk* OR "community garden?1" OR "food desert" OR "grocery store density" OR "fast food" OR "restaurant density" OR "healthy food availability" OR HFAI OR "nutrition environment measures" OR NEMS) |
| 43 | TS=((owner NEAR/2 (vehicle OR auto* OR car)) OR "alcohol outlet*" OR neighbo$rhood OR "housing quality" OR "urban environment" OR "inner cit*") |
| 44 | TS=((literate OR literacy) NEAR/2 (health OR English)) |
| 45 | TS=(degree NEAR/2 (university OR college OR advanced OR "high school")) |
| 46 | TS=(GED OR "high school graduate" OR "high school dropout" OR "high school completion") |
| 47 | TS=(educational* NEAR/2 (level$ OR status OR attainment OR achievement OR access)) |
| 48 | TS=("patient navigat*" OR "transcultural care" OR "culturally appropriate care" OR "culturally competent care" OR "cultural care" OR "cultural competenc*") |
| 49 | TS=((healthcare OR care OR provider OR "health services" OR insurance) NEAR/2 (access* OR availab*)) |
| 50 | #33 OR #34 OR #35 OR #36 OR #37 OR #38 OR #39 OR #40 OR #41 OR #42 OR #43 OR #44 OR #45 OR #46 OR #47 OR #48 OR #49 |
| 51 | #32 AND #50 |
| 52 | #51 AND LA=(English) |
| 53 | #52 NOT DT=(Meeting Abstract) |
| 54 | #53 AND CU=(USA) |
| 55 | #54 Timespan: 2020-01-01 to 2023-11-23 |
