# Supplementary material for: Associations of Social and Demographic Factors on the Outcomes of Ocular Melanoma and Other Adult Ocular Neoplasms in the United States: A Systematic Review
Source: Cochrane Evid Synth Methods. 2026 Mar 10;4(2):e70075. doi: 10.1002/cesm.70075 (PMC12977123; doi:10.1002/cesm.70075)
Supplement: Supplementary file 2 — Appendix B: Combined SRDR+ and qualtrics data extraction forms. [file CESM-4-e70075-s002.PDF]

Appendix B: Combined SRDR+ and Qualtrics Data Extraction Forms

☑ Default question

1. Extractor?

- ○ Vijay Joshi
- ○ Louis Leslie
- ○ Natalia Dellavalle
- ○ Other, specify: ____

2. Adjudicator

- ○ Vijay Joshi
- ○ Louis Leslie
- ○ Natalia Dellavalle
- ○ Other, specify: ____

3. Adjudication complete?

-- Select --

4. Study ID?

Author and year of paper

____

5. Study design?

Pick one, specify if needed

- ○ Case-control
- ○ Cohort (prospective or retrospective)
- ○ Controlled trial (not randomized)
- ○ Cross-sectional
- ○ Randomized controlled trial
- ○ Other, Specify: ______

6. Study funding source(s) verbatim:

Include the exact author statement on funding source, if present

Include quotes

______

7. Study funding source(s) category:

Include all that apply

- ☐ Industry
- ☐ Government or institution (non-institution)
- ☐ Authors stated that study was not funded
- ☐ Not reported
- ☐ Other, specify: ______

8. Study years

Format as "YYYY to YYYY"

If no given study timeline, input range of diagnosis (e.g. if study looked at retinoblastoma diagnoses between 2000-2014, put “2000 to 2014”)

______

9. Study Setting?

Pick the setting in which the study was conducted

	If other, specify here:
◯ Case-control<br>◯ Cohort (prospective or retrospective)<br>◯ Controlled trial (not randomized)<br>◯ Cross-sectional<br>◯ Randomized controlled trial<br>◯ Other, specify:	

6. Was the sample representative (cross-sectional studies)? Was the study sample free of selection bias (other study designs)?

	Rationale?
-- Select --	

7. Is the assessment of exposure free from information bias?

	Rationale?
-- Select --	

8. Is the assessment of outcome free from information bias?

	Rationale?
-- Select --	

9. Is the association free from confounding?

	Rationale?
-- Select --	

10. Overall risk of bias?

TBD how to exactly choose low/moderate/high

	Rationale?
-- Select --	

- [ ] Clinic, hospital, or health system based, specify
- [ ] Household
- [ ] Neighborhood
- [ ] Population based survey (e.g. SEER)
- [ ] School based
- [ ] Work or office based
- [ ] Other, specify:

10. Total sample size

NR if not reported

11. Type of ocular cancer studied?

Pick from the drop down selections. If other, specify

- [ ] Unilateral retinoblastoma
- [ ] Bilateral retinoblastoma
- [ ] Uveal melanoma
- [ ] Ocular surface neoplasia
- [ ] Other, specify:

12. Domains of SDOH studied?

Check all

- [ ] Economic stability
- [ ] Education access and quality
- [ ] Healthcare access and quality
- [ ] Neighborhood and built environment
- [ ] Social and community context

13. Specific SDOH constructs (A) - Economic stability

- [ ] Debt, specify (verbatim) ______
- [ ] Employment, specify verbatim ______
- [ ] Expenses, specify verbatim ______
- [ ] Food insecurity, specify verbatim ______
- [ ] Housing instability, specify verbatim ______
- [ ] Income instability, specify verbatim ______
- [ ] Income support, specify verbatim ______
- [ ] Medical bills, specify verbatim ______
- [ ] Poverty and concentrated poverty, specify verbatim ______

14. Specific SDOH constructs (B) - Education access and quality

Select the specific construct(s) studied and include the verbatim description of construct

- [ ] Community educational attainment, specify (verbatim) ______
- [ ] Early childhood education, specify (verbatim) ______
- [ ] High school graduation, specify (verbatim) ______
- [ ] Higher education, specify (verbatim) ______
- [ ] Language and literacy skills, specify (verbatim) ______
- [ ] Vocational training , specify (verbatim) ______

15. Specific SDOH constructs (C) - Healthcare access and quality

Select the specific construct(s) studied and include the verbatim description of construct

- [ ] Access to primary care, specify (verbatim)
- [ ] Affordability, specify (verbatim)
- [ ] Cost, specify (verbatim)
- [ ] Financial toxicity of healthcare treatments, specify (verbatim)
- [ ] Geographical access, proximity, and catchment area, specify (verbatim)
- [ ] Health insurance coverage, specify (verbatim)
- [ ] Health literacy, specify (verbatim)
- [ ] Health policy, specify (verbatim)
- [ ] Provider availability, specify (verbatim)
- [ ] Provider and ancillary personnel (e.g., technicians, receptionists) linguistic and cultural competency and empathy, specify (verbatim)
- [ ] Quality of care, specify (verbatim)
- [ ] Telehealth, telemedicine, and mobile health, specify (verbatim)
- [ ] Treatment adherence, specify (verbatim)

16. Specific SDOH constructs (D) - Neighborhood and built environment

Select the specific construct(s) studied and include the verbatim description of construct

- [ ] Access to healthy foods to support healthy eating, food swamps, and food deserts, specify (verbatim)
- [ ] Broadband, Internet, and Wi-Fi access, specify (verbatim)
- [ ] Library access, specify (verbatim)
- [ ] Census tract, specify (verbatim)
- [ ] Environmental conditions (e.g., air or water quality), specify (verbatim)
- [ ] Housing quality and pest infestation, specify (verbatim)
- [ ] Parks and playgrounds, specify (verbatim)
- [ ] Safety, specify (verbatim)
- [ ] Transportation, specify (verbatim)
- [ ] Walkability, specify (verbatim)
- [ ] Maintenance of streets and sidewalks, specify (verbatim)
- [ ] Graffiti, loose trash, specify (verbatim)
- [ ] Abandoned properties, specify (verbatim)
- [ ] Proportion of home rental vs ownership, specify (verbatim)
- [ ] Undesirable facilities in or adjacent to the neighborhood, specify (verbatim)
- [ ] Evidence of drug use/users, specify (verbatim)
- [ ] Fire department facility and others in the neighborhood, specify (verbatim)
- [ ] Nearest pharmacy, specify (verbatim)

17. Specific SDOH constructs (E) - Social and community context

Select the specific construct(s) studied and include the verbatim description of construct

- [ ] Adverse childhood experiences, specify (verbatim)
- [ ] Bias, specify (verbatim)
- [ ] Civic engagement and participation, specify (verbatim)
- [ ] Community engagement, specify (verbatim)
- [ ] Discrimination, specify (verbatim)
- [ ] Exposure to violence and trauma, specify (verbatim)
- [ ] Incarceration and criminal justice system, specify (verbatim)
- [ ] Racial and ethnic residential segregation, specify (verbatim)
- [ ] Racism, specify (verbatim)
- [ ] Sense of community, specify (verbatim)
- [ ] Social capital and networks, specify (verbatim)
- [ ] Social cohesion and integration, specify (verbatim)
- [ ] Social isolation, specify (verbatim)
- [ ] Social support and support systems, specify (verbatim)
- [ ] Social vulnerability, specify (verbatim)
- [ ] Trust, specify (verbatim)
- [ ] Presence of police personnel, specify (verbatim)
- [ ] Race/ethnicity, specify (verbatim)

18. Specific construct(s)?

Enter the reported study variable(s) corresponding to all studied SDOH domains. Input in in list form separated by semicolon.

19. Measurement of SDOH variable? Verbatim

Enter the method of measuring the variable, verbatim if possible

20. Measurement of SDOH variable?

[illegible]

- [ ] Self report
- [ ] Instrument, specify: ____
- [ ] Expert interview, specify experts ____
- [ ] Scale, specify: ____
- [ ] Other, specify: ____

21. Notes

Enter any relevant notes or questions here

Arm Suggestions

You may suggest Arms for extractors to choose from. Suggesting Arm helps keep data consistent among data extractions.
Friendly Helper

List of Suggested Arms

Name	Description	
Overall single group	No comparison arm	Edit<br>Remove
Cases	People with the outcome/condition, specify: _______________	Edit<br>Remove
Controls	People without the outcome/condition, specify: _______________	Edit<br>Remove
Exposed group	People with an exposure/intervention of interest, specify: _______________	Edit<br>Remove
Unexposed group	People without an exposure/intervention of interest, specify: _______________	Edit<br>Remove

Add Arm Suggestion

* Name

Description

Save

Select Key Question to work on

☑ Default question

1. Age (cases)

Extract the mean (or median) and std. deviation (IQR) for Age for all groups.

If categories are provided instead, report the numbers and metrics for age in each group (e.g., 8-16 months = 20; 17-24 months = 32; etc.)
Overall single group:

	Select the measures	Values
Estimate	○ Mean<br>○ Median<br>○ Categorical, specify categories: ___<br>○ Not reported	___
Imprecision	○ Standard deviation<br>○ Interquartile Range<br>○ Not applicable<br>○ Not reported	___

2. Reported parents ages

Does the report provide the ages for mothers, fathers, or both
Overall single group:

☐ Mothers
☐ Fathers
☐ Not reported

3. Age (mothers)

Extract the mean (or median) and std. deviation (IQR) for Age for all groups.

If categorical distribution, extract values for each group separated by semicolons (e.g., 18-24 = 20; 25-35 = 32; etc.)
Overall single group:

	Select the measures	Values
Estimate	◯ Mean<br>◯ Median<br>◯ Categories, specify groupings:	
Imprecision	◯ Standard deviation<br>◯ Interquartile Range<br>◯ Not applicable	

4. Age (fathers)

Extract the mean (or median) and std. deviation (IQR) for Age for all groups.

If categorical distribution, extract values for each group separated by semicolons (e.g., 18-24 = 20; 25-35 = 32; etc.)
Overall single group:

	Select the measures	Values
Estimate	◯ Mean<br>◯ Median<br>◯ Categories, specify groupings:	
Imprecision	◯ Standard deviation<br>◯ Interquartile Range<br>◯ Not applicable	

5. Sex of cases

Extract the distribution of sex for the cases
Overall single group:

Male	Female

6. Ethnicity of parent(s) and/or children participants

Extract the distribution of ethnicity for parent(s).

If not reported, write "NR"
Overall single group:

	Mother	Father	Non-specified (parent)	Case
Non-hispanic white				
Black				
Hispanic				
Asian and Pacific Islander				
Other, specify:				

Outcome Suggestions

You may suggest Outcomes for extractors to choose from. Suggesting Outcome helps keep data consistent among data extractions.

Friendly Helper

List of Suggested Outcomes

Type	Domain	Specific measurement (i.e., tool/definition/specific outcome)	Timepoint(s)	
Categorical	Retinoblastoma	Any association with development of retinoblastoma (risk ratio, odds ratio, hazard ratio)	• N/A	Edit Remove
Categorical	Uveal melanoma	Any association with development of uveal melanoma (risk ratio, odds ratio, hazard ratio)	• N/A	Edit Remove
Categorical	Ocular surface neoplasia	Any association with development of ocular surface neoplasia (risk ratio, odds ratio, hazard ratio)	• N/A	Edit Remove
Categorical	Squamous cell carcinoma of the conjunctiva	Any association with development of Squamous cell carcinoma of the conjunctiva (risk ratio, odds ratio, hazard ratio)	• N/A	Edit Remove
Categorical	Other ocular cancer	Any association with development of other ocular cancer (risk ratio, odds ratio, hazard ratio)	• N/A	Edit Remove

Add Outcome Suggestion

* Suggest Type of Outcome

* Suggest Domain

Suggest Specific measurement (i.e., tool/definition/specific outcome)

Suggest Timepoint(s) to associate with this Outcome. If you cannot find an existing Timepoint to associate, click here to

Select Key Question to work on

- [x] Default question

1. 1. Diagnosis criteria

Copy and paste verbatim from the paper

Retinoblastoma:

2. EXTRACT ALL OUTCOMES AND ESTIMATES FOR ASSOCIATIONS IN QUALTRICS

Use the following link to extract associations. Mark whether this has been done for each outcome.

https://ucdenver.co1.qualtrics.com/jfe/form/SV_9KOh5uFI9ZoVBqe

Retinoblastoma:

- ( ) Completed

Qualtrics - SDOH and Ocular cancer

Survey Flow

Block: Default Question Block (6 Questions)
Standard: Block 2 (2 Questions)
Standard: Association 1 (2 Questions)
Standard: Association 2 (2 Questions)
Standard: Association 3 (2 Questions)
Standard: Association 4 (2 Questions)
Standard: Association 5 (2 Questions)
Standard: Association 6 (2 Questions)
Standard: Association 7 (2 Questions)
Standard: Association 8 (2 Questions)
Standard: Association 9 (2 Questions)
Standard: Association 10 (2 Questions)
Standard: Association 11 (2 Questions)
Standard: Association 12 (2 Questions)
Standard: Association 13 (2 Questions)
Standard: Association 14 (2 Questions)
Standard: Association 15 (2 Questions)
Standard: Association 16 (2 Questions)
Standard: Association 17 (2 Questions)
Standard: Association 18 (2 Questions)
Standard: Association 19 (2 Questions)
Standard: Association 20 (2 Questions)

Page Break

Start of Block: Default Question Block

Q1 Q1. SDOH Domains
Select all that apply.

- ☐ Economic stability (1)
- ☐ Education access and quality (2)
- ☐ Healthcare access and quality (3)
- ☐ Neighborhood and built environment (4)
- ☐ Social and community context (5)

Display This Question:

If Q1 = 1

Q2 Q2a. Economic stability

Select all that apply. Select the specific construct(s) studied and include the description of construct as defined in the study verbatim.

- ▢ Debt (1) ____
- ▢ Employment (2) ____
- ▢ Expenses (3) ____
- ▢ Food insecurity (4) ____
- ▢ Housing instability (5) ____
- ▢ Income instability (6) ____
- ▢ Income support (7) ____
- ▢ Medical bills (8) ____
- ▢ Poverty and concentrated poverty (9) ____
- ▢ Other (10) ____

Display This Question:

If Q1 = 2

Q3 Q2b. Education access and quality

Select all that apply. Select the specific construct(s) studied and include the description of construct as defined in the study verbatim.

- ▢ Community educational attainment (1) ____
- ▢ Early childhood education (2) ____
- ▢ High school graduation (3) ____
- ▢ Higher education (4) ____
- ▢ Language and literacy skills (5) ____
- ▢ Vocational training (6) ____
- ▢ Other (7)

Display This Question:

If Q1 = 3

Q4 Q2c. Healthcare access and quality
Select all that apply. Select the specific construct(s) studied and include the description of construct as defined in the study verbatim.

- ☐ Access to primary care (1) ____
- ☐ Affordability (2) ____
- ☐ Cost (3) ____
- ☐ Financial toxicity of healthcare treatments (4) ____
- ☐ Geographical access, proximity, and catchment area (5) ____
- ☐ Health insurance coverage (6) ____
- ☐ Health literacy (7) ____
- ☐ Health policy (8) ____
- ☐ Provider availability (9) ____
- ☐ Provider and ancillary personnel (e.g., technicians, receptionists) linguistic and cultural competency and empathy (10) ____

☐ Quality of care (11) ____

☐ Telehealth, telemedicine, and mobile health (12) ____

☐ Treatment adherence (13) ____

☐ Other (14) ____

Display This Question:

If Q1 = 4

Q5 Q2d. Neighborhood and built environment
Select all that apply. Select the specific construct(s) studied and include the description of construct as defined in the study verbatim.

- ☐ Access to healthy foods to support healthy eating, food swamps, and food deserts (1) ____
- ☐ Broadband, Internet, and Wi-Fi access (2) ____
- ☐ Library access (3) ____
- ☐ Census tract (4) ____
- ☐ Environmental conditions (e.g., air or water quality) (5) ____
- ☐ Housing quality and pest infestation (6) ____
- ☐ Parks and playgrounds (7) ____
- ☐ Safety (8) ____
- ☐ Transportation (9) ____
- ☐ Walkability (10) ____

- [ ] Maintenance of streets and sidewalks (11) ____

- [ ] Graffiti, loose trash (12) ____

- [ ] Abandoned properties (13) ____

- [ ] Proportion of home rental vs ownership (14) ____

- [ ] Undesirable facilities in or adjacent to the neighborhood (15) ____

- [ ] Evidence of drug use/users (16) ____

- [ ] Fire department facility and others in the neighborhood (17) ____

- [ ] Nearest pharmacy (18) ____

- [ ] Other (19) ____

Display This Question:

If Q1 = 5

Q6 Q2e. Social and community context

Select all that apply. Select the specific construct(s) studied and include the description of construct as defined in the study verbatim.

- ▢ Adverse childhood experiences (1) ____
- ▢ Bias (2) ____
- ▢ Civic engagement and participation (3) ____
- ▢ Community engagement (4)
- ▢ Discrimination (5) ____
- ▢ Exposure to violence and trauma (6) ____
- ▢ Incarceration and criminal justice system (7) ____
- ▢ Racial and ethnic residential segregation (8) ____
- ▢ Racism (9) ____
- ▢ Sense of community (10) ____
- ▢ Social capital and networks (11) ____

- [ ] Social cohesion and integration (12) ____
- [ ] Social isolation (13) ____
- [ ] Social support and support systems (14) ____
- [ ] Social vulnerability (15) ____
- [ ] Trust (16) ____
- [ ] Presence of police personnel (17) ____
- [ ] Other (18) ____

End of Block: Default Question Block

Start of Block: Block 2

Q9 How many associations are described in the study?

▼ 1 (1) ... More than 20 (21)

Q10 For each social determinant association, what is the exposure-outcome assessed?

	Exposure	Outcome	Which estimate is given?		
	Specify the exposure (1)	Specify the outcome (1)	Adjusted (quantitative) (1)	Unadjusted (quantitative) (2)	Descriptive (qualitative) (3)

Association 1 (1)			☐	☐	☐
Association 2 (2)			☐	☐	☐
Association 3 (3)			☐	☐	☐
Association 4 (4)			☐	☐	☐
Association 5 (5)			☐	☐	☐

Association 6 (6)					
Association 7 (7)					
Association 8 (8)					
Association 9 (9)					
Association 10 (10)					

Association 11 (11)			☐	☐	☐
Association 12 (12)			☐	☐	☐
Association 13 (13)			☐	☐	☐
Association 14 (14)			☐	☐	☐
Association 15 (15)			☐	☐	☐

Association 16 (16)			▢	▢	▢
Association 17 (17)			▢	▢	▢
Association 18 (18)			▢	▢	▢
Association 19 (19)			▢	▢	▢
Association 20 (20)			▢	▢	▢

End of Block: Block 2

Start of Block: Association 1

Q11 Association 1 - Quantitative: ${Q10%231/ChoiceTextEntryValue/1/1} - ${Q10%232/ChoiceTextEntryValue/1/1}

Fill in each row (adjusted/unadjusted) as reported. If "Other" effect, specify in the "effect estimate" column. For the adjusted estimate, specify all adjustment variables.

	Effect measure					Effect estimate	95% CI	P-value	Adjusted variables	Interpretation
	Beta (1)	OR (2)	RR (3)	HR (4)	Other (5)	(e.g., 1.23) (1)	(e.g., 1.15 - 1.36) (1)	(e.g., 0.001) (1)	Specify all variables adjusted for in analyses (separate by semicolon) (1)	Summarize the direction and effect of the association (1)

Adjusted association (1)	○	○	○	○	○					
Unadjusted association (2)	○	○	○	○	○					

Display This Question:

If Q10#3 = 1 [3]

Q17 Association 1 - Qualitative: ${Q10%231/ChoiceTextEntryValue/1/1} - ${Q10%232/ChoiceTextEntryValue/1/1}

Copy and paste the description of the association.

____

End of Block: Association 1

Start of Block: Association 2

Display This Question:

If Q10#3 = 2 [1]

Or Q10#3 = 2 [2]

Q18 Association 2 - Quantitative: ${Q10%231/ChoiceTextEntryValue/2/1} - ${Q10%232/ChoiceTextEntryValue/2/1}

Fill in each row (adjusted/unadjusted) as reported. If "Other" effect, specify in the "effect estimate" column. For the adjusted estimate, specify all adjustment variables.

	Effect measure					Effect estimate	95% CI	P-value	Adjusted variables	Interpretation
	Beta (1)	OR (2)	RR (3)	HR (4)	Other (5)	(e.g., 1.23) (1)	(e.g., 1.15 - 1.36) (1)	(e.g., 0.001) (1)	Specify all variables adjusted for in analyses (separate by semicolon) (1)	Summarize the direction and effect of the association (1)

Adjusted association (1)	○	○	○	○	○					
Unadjusted association (2)	○	○	○	○	○					

Display This Question:

If Q10#3 = 2 [3]

Q19 Association 2 - Qualitative: ${Q10%231/ChoiceTextEntryValue/2/1} - ${Q10%232/ChoiceTextEntryValue/2/1}

Copy and paste the description of the association.

____

End of Block: Association 2

Start of Block: Association 3

Display This Question:

If Q10#3 = 3 [1]

Or Q10#3 = 3 [2]

Q20 Association 3 - Quantitative: ${Q10%231/ChoiceTextEntryValue/3/1} - ${Q10%232/ChoiceTextEntryValue/3/1}

Fill in each row (adjusted/unadjusted) as reported. If "Other" effect, specify in the "effect estimate" column. For the adjusted estimate, specify all adjustment variables.

	Effect measure					Effect estimate	95% CI	P-value	Adjusted variables	Interpretation
	Beta (1)	OR (2)	RR (3)	HR (4)	Other (5)	(e.g., 1.23) (1)	(e.g., 1.15 - 1.36) (1)	(e.g., 0.001) (1)	Specify all variables adjusted for in analyses (separate by semicolon) (1)	Summarize the direction and effect of the association (1)

Adjusted association (1)	○	○	○	○	○					
Unadjusted association (2)	○	○	○	○	○					

Display This Question:

If Q10#3 = 3 [3]

Q21 Association 3 - Qualitative: ${Q10%231/ChoiceTextEntryValue/3/1} - ${Q10%232/ChoiceTextEntryValue/3/1}

Copy and paste the description of the association.

____

End of Block: Association 3

Start of Block: Association 4

Display This Question:

If Q10#3 = 4 [1]

Or Q10#3 = 4 [2]

Q22 Association 4 - Quantitative: ${Q10%231/ChoiceTextEntryValue/4/1} - ${Q10%232/ChoiceTextEntryValue/4/1}

Fill in each row (adjusted/unadjusted) as reported. If "Other" effect, specify in the "effect estimate" column. For the adjusted estimate, specify all adjustment variables.

	Effect measure					Effect estimate	95% CI	P-value	Adjusted variables	Interpretation
	Beta (1)	OR (2)	RR (3)	HR (4)	Other (5)	(e.g., 1.23) (1)	(e.g., 1.15 - 1.36) (1)	(e.g., 0.001) (1)	Specify all variables adjusted for in analyses (separate by semicolon) (1)	Summarize the direction and effect of the association (1)

Adjusted association (1)	○	○	○	○	○				
Unadjusted association (2)	○	○	○	○	○				

Display This Question:

If Q10#3 = 4 [3]

Q23 Association 4 - Qualitative: ${Q10%231/ChoiceTextEntryValue/4/1} - ${Q10%232/ChoiceTextEntryValue/4/1}

Copy and paste the description of the association.

____

End of Block: Association 4

Start of Block: Association 5

Display This Question:

If Q10#3 = 5 [1]

Or Q10#3 = 5 [2]

Q24 Association 5 - Quantitative: ${Q10%231/ChoiceTextEntryValue/5/1} - ${Q10%232/ChoiceTextEntryValue/5/1}

Fill in each row (adjusted/unadjusted) as reported. If "Other" effect, specify in the "effect estimate" column. For the adjusted estimate, specify all adjustment variables.

	Effect measure					Effect estimate	95% CI	P-value	Adjusted variables	Interpretation
	Beta (1)	OR (2)	RR (3)	HR (4)	Other (5)	(e.g., 1.23) (1)	(e.g., 1.15 - 1.36) (1)	(e.g., 0.001) (1)	Specify all variables adjusted for in analyses (separate by semicolon) (1)	Summarize the direction and effect of the association (1)

Adjusted association (1)	○	○	○	○	○				
Unadjusted association (2)	○	○	○	○	○				

Display This Question:

If Q10#3 = 5 [3]

Q25 Association 5 - Qualitative: ${Q10%231/ChoiceTextEntryValue/5/1} - ${Q10%232/ChoiceTextEntryValue/5/1}

Copy and paste the description of the association.

____

End of Block: Association 5

Start of Block: Association 6

Display This Question:

If Q10#3 = 6 [1]

Or Q10#3 = 6 [2]

Q26 Association 6 - Quantitative: ${Q10%231/ChoiceTextEntryValue/6/1} - ${Q10%232/ChoiceTextEntryValue/6/1}

Fill in each row (adjusted/unadjusted) as reported. If "Other" effect, specify in the "effect estimate" column. For the adjusted estimate, specify all adjustment variables.

	Effect measure					Effect estimate	95% CI	P-value	Adjusted variables	Interpretation
	Beta (1)	OR (2)	RR (3)	HR (4)	Other (5)	(e.g., 1.23) (1)	(e.g., 1.15 - 1.36) (1)	(e.g., 0.001) (1)	Specify all variables adjusted for in analyses (separate by semicolon) (1)	Summarize the direction and effect of the association (1)

Adjusted association (1)	○	○	○	○	○				
Unadjusted association (2)	○	○	○	○	○				

Display This Question:

If Q10#3 = 6 [3]

Q27 Association 6 - Qualitative: ${Q10%231/ChoiceTextEntryValue/6/1} - ${Q10%232/ChoiceTextEntryValue/6/1}

Copy and paste the description of the association.

____

End of Block: Association 6

Start of Block: Association 7

Display This Question:

If Q10#3 = 7 [1]

Or Q10#3 = 7 [2]

Q28 Association 7 - Quantitative: ${Q10%231/ChoiceTextEntryValue/7/1} - ${Q10%232/ChoiceTextEntryValue/7/1}

Fill in each row (adjusted/unadjusted) as reported. If "Other" effect, specify in the "effect estimate" column. For the adjusted estimate, specify all adjustment variables.

	Effect measure					Effect estimate	95% CI	P-value	Adjusted variables	Interpretation
	Beta (1)	OR (2)	RR (3)	HR (4)	Other (5)	(e.g., 1.23) (1)	(e.g., 1.15 - 1.36) (1)	(e.g., 0.001) (1)	Specify all variables adjusted for in analyses (separate by semicolon) (1)	Summarize the direction and effect of the association (1)

Adjusted association (1)	○	○	○	○	○					
Unadjusted association (2)	○	○	○	○	○					

Display This Question:

If Q10#3 = 7 [3]

Q29 Association 7 - Qualitative: ${Q10%231/ChoiceTextEntryValue/7/1} - ${Q10%232/ChoiceTextEntryValue/7/1}

Copy and paste the description of the association.

____

End of Block: Association 7

Start of Block: Association 8

Display This Question:

If Q10#3 = 8 [1]

Or Q10#3 = 8 [2]

Q30 Association 8 - Quantitative: ${Q10%231/ChoiceTextEntryValue/8/1} - ${Q10%232/ChoiceTextEntryValue/8/1}

Fill in each row (adjusted/unadjusted) as reported. If "Other" effect, specify in the "effect estimate" column. For the adjusted estimate, specify all adjustment variables.

	Effect measure					Effect estimate	95% CI	P-value	Adjusted variables	Interpretation
	Beta (1)	OR (2)	RR (3)	HR (4)	Other (5)	(e.g., 1.23) (1)	(e.g., 1.15 - 1.36) (1)	(e.g., 0.001) (1)	Specify all variables adjusted for in analyses (separate by semicolon) (1)	Summarize the direction and effect of the association (1)

Adjusted association (1)	○	○	○	○	○					
Unadjusted association (2)	○	○	○	○	○					

Display This Question:

If Q10#3 = 8 [3]

Q31 Association 8 - Qualitative: ${Q10%231/ChoiceTextEntryValue/8/1} - ${Q10%232/ChoiceTextEntryValue/8/1}

Copy and paste the description of the association.

____

End of Block: Association 8

Start of Block: Association 9

Display This Question:

If Q10#3 = 9 [1]

Or Q10#3 = 9 [2]

Q32 Association 9 - Quantitative: ${Q10%231/ChoiceTextEntryValue/9/1} - ${Q10%232/ChoiceTextEntryValue/9/1}

Fill in each row (adjusted/unadjusted) as reported. If "Other" effect, specify in the "effect estimate" column. For the adjusted estimate, specify all adjustment variables.

	Effect measure					Effect estimate	95% CI	P-value	Adjusted variables	Interpretation
	Beta (1)	OR (2)	RR (3)	HR (4)	Other (5)	(e.g., 1.23) (1)	(e.g., 1.15 - 1.36) (1)	(e.g., 0.001) (1)	Specify all variables adjusted for in analyses (separate by semicolon) (1)	Summarize the direction and effect of the association (1)

Adjusted association (1)	○	○	○	○	○					
Unadjusted association (2)	○	○	○	○	○					

Display This Question:

If Q10#3 = 9 [3]

Q33 Association 9 - Qualitative: ${Q10%231/ChoiceTextEntryValue/9/1} - ${Q10%232/ChoiceTextEntryValue/9/1}

Copy and paste the description of the association.

____

End of Block: Association 9

Start of Block: Association 10

Display This Question:

If Q10#3 = 10 [1]

Or Q10#3 = 10 [2]

Q34 Association 10 - Quantitative: ${Q10%231/ChoiceTextEntryValue/10/1} - ${Q10%232/ChoiceTextEntryValue/10/1}

Fill in each row (adjusted/unadjusted) as reported. If "Other" effect, specify in the "effect estimate" column. For the adjusted estimate, specify all adjustment variables.

	Effect measure					Effect estimate	95% CI	P-value	Adjusted variables	Interpretation
	Beta (1)	OR (2)	RR (3)	HR (4)	Other (5)	(e.g., 1.23) (1)	(e.g., 1.15 - 1.36) (1)	(e.g., 0.001) (1)	Specify all variables adjusted for in analyses (separate by semicolon) (1)	Summarize the direction and effect of the association (1)

Adjusted association (1)	○	○	○	○	○					
Unadjusted association (2)	○	○	○	○	○					

Display This Question:

If Q10#3 = 10 [3]

Q35 Association 10 - Qualitative: ${Q10%231/ChoiceTextEntryValue/10/1} - ${Q10%232/ChoiceTextEntryValue/10/1}

Copy and paste the description of the association.

____

End of Block: Association 10

Start of Block: Association 11

Display This Question:

If Q10#3 = 11 [1]

Or Q10#3 = 11 [2]

Q36 Association 11 - Quantitative: ${Q10%231/ChoiceTextEntryValue/11/1} - ${Q10%232/ChoiceTextEntryValue/11/1}

Fill in each row (adjusted/unadjusted) as reported. If "Other" effect, specify in the "effect estimate" column. For the adjusted estimate, specify all adjustment variables.

	Effect measure					Effect estimate	95% CI	P-value	Adjusted variables	Interpretation
	Beta (1)	OR (2)	RR (3)	HR (4)	Other (5)	(e.g., 1.23) (1)	(e.g., 1.15 - 1.36) (1)	(e.g., 0.001) (1)	Specify all variables adjusted for in analyses (separate by semicolon) (1)	Summarize the direction and effect of the association (1)

Adjusted association (1)	○	○	○	○	○					
Unadjusted association (2)	○	○	○	○	○					

Display This Question:

If Q10#3 = 11 [3]

Q37 Association 11 - Qualitative: ${Q10%231/ChoiceTextEntryValue/11/1} - ${Q10%232/ChoiceTextEntryValue/11/1}

Copy and paste the description of the association.

____

End of Block: Association 11

Start of Block: Association 12

Display This Question:

If Q10#3 = 12 [1]

Or Q10#3 = 12 [2]

Q44 Association 12 - Quantitative: ${Q10%231/ChoiceTextEntryValue/12/1} - ${Q10%232/ChoiceTextEntryValue/12/1}

Fill in each row (adjusted/unadjusted) as reported. If "Other" effect, specify in the "effect estimate" column. For the adjusted estimate, specify all adjustment variables.

	Effect measure					Effect estimate	95% CI	P-value	Adjusted variables	Interpretation
	Beta (1)	OR (2)	RR (3)	HR (4)	Other (5)	(e.g., 1.23) (1)	(e.g., 1.15 - 1.36) (1)	(e.g., 0.001) (1)	Specify all variables adjusted for in analyses (separate by semicolon) (1)	Summarize the direction and effect of the association (1)

Adjusted association (1)	○	○	○	○	○					
Unadjusted association (2)	○	○	○	○	○					

Display This Question:

If Q10#3 = 12 [3]

Q45 Association 12 - Qualitative: ${Q10%231/ChoiceTextEntryValue/12/1} - ${Q10%232/ChoiceTextEntryValue/12/1}

Copy and paste the description of the association.

____

End of Block: Association 12

Start of Block: Association 13

Display This Question:

If Q10#3 = 13 [1]

Or Q10#3 = 13 [2]

Q46 Association 13 - Quantitative: ${Q10%231/ChoiceTextEntryValue/13/1} - ${Q10%232/ChoiceTextEntryValue/13/1}

Fill in each row (adjusted/unadjusted) as reported. If "Other" effect, specify in the "effect estimate" column. For the adjusted estimate, specify all adjustment variables.

	Effect measure					Effect estimate	95% CI	P-value	Adjusted variables	Interpretation
	Beta (1)	OR (2)	RR (3)	HR (4)	Other (5)	(e.g., 1.23) (1)	(e.g., 1.15 - 1.36) (1)	(e.g., 0.001) (1)	Specify all variables adjusted for in analyses (separate by semicolon) (1)	Summarize the direction and effect of the association (1)

Adjusted association (1)	○	○	○	○	○					
Unadjusted association (2)	○	○	○	○	○					

Display This Question:

If Q10#3 = 13 [3]

Q47 Association 13 - Qualitative: ${Q10%231/ChoiceTextEntryValue/13/1} - ${Q10%232/ChoiceTextEntryValue/13/1}

Copy and paste the description of the association.

____

End of Block: Association 13

Start of Block: Association 14

Display This Question:

If Q10#3 = 14 [1]

Or Q10#3 = 14 [2]

Q48 Association 14 - Quantitative: ${Q10%231/ChoiceTextEntryValue/14/1} - ${Q10%232/ChoiceTextEntryValue/14/1}

Fill in each row (adjusted/unadjusted) as reported. If "Other" effect, specify in the "effect estimate" column. For the adjusted estimate, specify all adjustment variables.

	Effect measure					Effect estimate	95% CI	P-value	Adjusted variables	Interpretation
	Beta (1)	OR (2)	RR (3)	HR (4)	Other (5)	(e.g., 1.23) (1)	(e.g., 1.15 - 1.36) (1)	(e.g., 0.001) (1)	Specify all variables adjusted for in analyses (separate by semicolon) (1)	Summarize the direction and effect of the association (1)

Adjusted association (1)	o	o	o	o	o				
Unadjusted association (2)	o	o	o	o	o				

Display This Question:

If Q10#3 = 14 [3]

Q49 Association 14 - Qualitative: ${Q10%231/ChoiceTextEntryValue/14/1} - ${Q10%232/ChoiceTextEntryValue/14/1}

Copy and paste the description of the association.

____

End of Block: Association 14

Start of Block: Association 15

Display This Question:

If Q10#3 = 15 [1]

Or Q10#3 = 15 [2]

Q50 Association 15 - Quantitative: ${Q10%231/ChoiceTextEntryValue/15/1} - ${Q10%232/ChoiceTextEntryValue/15/1}

Fill in each row (adjusted/unadjusted) as reported. If "Other" effect, specify in the "effect estimate" column. For the adjusted estimate, specify all adjustment variables.

	Effect measure					Effect estimate	95% CI	P-value	Adjusted variables	Interpretation
	Beta (1)	OR (2)	RR (3)	HR (4)	Other (5)	(e.g., 1.23) (1)	(e.g., 1.15 - 1.36) (1)	(e.g., 0.001) (1)	Specify all variables adjusted for in analyses (separate by semicolon) (1)	Summarize the direction and effect of the association (1)

Adjusted association (1)	○	○	○	○	○				
Unadjusted association (2)	○	○	○	○	○				

Display This Question:

If Q10#3 = 15 [3]

Q51 Association 15 - Qualitative: ${Q10%231/ChoiceTextEntryValue/15/1} - ${Q10%232/ChoiceTextEntryValue/15/1}

Copy and paste the description of the association.

____

End of Block: Association 15

Start of Block: Association 16

Display This Question:

If Q10#3 = 16 [1]

Or Q10#3 = 16 [2]

Q52 Association 16 - Quantitative: ${Q10%231/ChoiceTextEntryValue/16/1} - ${Q10%232/ChoiceTextEntryValue/16/1}

Fill in each row (adjusted/unadjusted) as reported. If "Other" effect, specify in the "effect estimate" column. For the adjusted estimate, specify all adjustment variables.

	Effect measure					Effect estimate	95% CI	P-value	Adjusted variables	Interpretation
	Beta (1)	OR (2)	RR (3)	HR (4)	Other (5)	(e.g., 1.23) (1)	(e.g., 1.15 - 1.36) (1)	(e.g., 0.001) (1)	Specify all variables adjusted for in analyses (separate by semicolon) (1)	Summarize the direction and effect of the association (1)

Adjusted association (1)	○	○	○	○	○				
Unadjusted association (2)	○	○	○	○	○				

Display This Question:

If Q10#3 = 16 [3]

Q53 Association 16 - Qualitative: ${Q10%231/ChoiceTextEntryValue/16/1} - ${Q10%232/ChoiceTextEntryValue/16/1}

Copy and paste the description of the association.

____

End of Block: Association 16

Start of Block: Association 17

Display This Question:

If Q10#3 = 17 [1]

Or Q10#3 = 17 [2]

Q54 Association 17 - Quantitative: ${Q10%231/ChoiceTextEntryValue/17/1} - ${Q10%232/ChoiceTextEntryValue/17/1}

Fill in each row (adjusted/unadjusted) as reported. If "Other" effect, specify in the "effect estimate" column. For the adjusted estimate, specify all adjustment variables.

	Effect measure					Effect estimate	95% CI	P-value	Adjusted variables	Interpretation
	Beta (1)	OR (2)	RR (3)	HR (4)	Other (5)	(e.g., 1.23) (1)	(e.g., 1.15 - 1.36) (1)	(e.g., 0.001) (1)	Specify all variables adjusted for in analyses (separate by semicolon) (1)	Summarize the direction and effect of the association (1)

Adjusted association (1)	○	○	○	○	○				
Unadjusted association (2)	○	○	○	○	○				

Display This Question:

If Q10#3 = 17 [3]

Q55 Association 17 - Qualitative: ${Q10%231/ChoiceTextEntryValue/17/1} - ${Q10%232/ChoiceTextEntryValue/17/1}

Copy and paste the description of the association.

____

End of Block: Association 17

Start of Block: Association 18

Display This Question:

If Q10#3 = 18 [1]

Or Q10#3 = 18 [2]

Q56 Association 18 - Quantitative: ${Q10%231/ChoiceTextEntryValue/18/1} - ${Q10%232/ChoiceTextEntryValue/18/1}

Fill in each row (adjusted/unadjusted) as reported. If "Other" effect, specify in the "effect estimate" column. For the adjusted estimate, specify all adjustment variables.

	Effect measure					Effect estimate	95% CI	P-value	Adjusted variables	Interpretation
	Beta (1)	OR (2)	RR (3)	HR (4)	Other (5)	(e.g., 1.23) (1)	(e.g., 1.15 - 1.36) (1)	(e.g., 0.001) (1)	Specify all variables adjusted for in analyses (separate by semicolon) (1)	Summarize the direction and effect of the association (1)

Adjusted association (1)	○	○	○	○	○					
Unadjusted association (2)	○	○	○	○	○					

Display This Question:

If Q10#3 = 18 [3]

Q57 Association 18 - Qualitative: ${Q10%231/ChoiceTextEntryValue/18/1} - ${Q10%232/ChoiceTextEntryValue/18/1}

Copy and paste the description of the association.

____

End of Block: Association 18

Start of Block: Association 19

Display This Question:

If Q10#3 = 19 [1]

Or Q10#3 = 19 [2]

Q58 Association 19 - Quantitative: ${Q10%231/ChoiceTextEntryValue/19/1} - ${Q10%232/ChoiceTextEntryValue/19/1}

Fill in each row (adjusted/unadjusted) as reported. If "Other" effect, specify in the "effect estimate" column. For the adjusted estimate, specify all adjustment variables.

	Effect measure					Effect estimate	95% CI	P-value	Adjusted variables	Interpretation
	Beta (1)	OR (2)	RR (3)	HR (4)	Other (5)	(e.g., 1.23) (1)	(e.g., 1.15 - 1.36) (1)	(e.g., 0.001) (1)	Specify all variables adjusted for in analyses (separate by semicolon) (1)	Summarize the direction and effect of the association (1)

Adjusted association (1)	○	○	○	○	○				
Unadjusted association (2)	○	○	○	○	○				

Display This Question:

If Q10#3 = 19 [3]

Q59 Association 19 - Qualitative: ${Q10%231/ChoiceTextEntryValue/19/1} - ${Q10%232/ChoiceTextEntryValue/19/1}

Copy and paste the description of the association.

____

End of Block: Association 19

Start of Block: Association 20

Display This Question:

If Q10#3 = 20 [1]

Or Q10#3 = 20 [2]

Q60 Association 20 - Quantitative: ${Q10%231/ChoiceTextEntryValue/20/1} - ${Q10%232/ChoiceTextEntryValue/20/1}

Fill in each row (adjusted/unadjusted) as reported. If "Other" effect, specify in the "effect estimate" column. For the adjusted estimate, specify all adjustment variables.

	Effect measure					Effect estimate	95% CI	P-value	Adjusted variables	Interpretation
	Beta (1)	OR (2)	RR (3)	HR (4)	Other (5)	(e.g., 1.23) (1)	(e.g., 1.15 - 1.36) (1)	(e.g., 0.001) (1)	Specify all variables adjusted for in analyses (separate by semicolon) (1)	Summarize the direction and effect of the association (1)

Adjusted association (1)	○	○	○	○	○				
Unadjusted association (2)	○	○	○	○	○				

Display This Question:

If Q10#3 = 20 [3]

Q61 Association 20 - Qualitative: ${Q10%231/ChoiceTextEntryValue/20/1} - ${Q10%232/ChoiceTextEntryValue/20/1}

Copy and paste the description of the association.

____

End of Block: Association 20
